# Chemical genetic screens combine target dependence with efficacy: discovery of a MAP3K1 degrader (HBC-8) with anticancer activities

**DOI:** 10.1186/s43556-026-00632-z

**Published:** 2026-10-28

**Authors:** Lelisse T. Umeta, Jayapal Reddy Mallareddy, Sarbjit Singh, Karan Vishwakarma, Smitha Kizhake, Wangbin Wu, Amritha G. Kizhake, Lidia Boghean, Suchita Vishwakarma, Mihir Kachhia, A. J. Sallee, Vaishnavi Sunil Joshi, Katelyn L. O’Neill, Lisa Humphrey-Brattain, Linda Chee, Matthew G. Rees, Melissa M. Ronan, Jennifer A. Roth, Andrew Chatr-aryamontri, Thierry Bertomeu, Kabhilan Mohan, Yuanhong Chen, Kyle Hewitt, Grinu Mathew, Adrian Black, Gargi Ghosal, Xu Luo, Jixin Dong, Nicholas T. Woods, Amarnath Natarajan

**Affiliations:** 1 Division of Pharmaceutical Sciences, James L. Winkle College of Pharmacy, University of Cincinnati, Cincinnati, OH 45267 USA; 2 Eppley Institute for Cancer Research, University of Nebraska Medical Center, Omaha, NE 68198 USA; 3 Department of Pharmaceutical Sciences, University of Nebraska Medical Center, Omaha, NE 68198 USA; 4 Department of Genetics, Cell Biology and Anatomy, University of Nebraska Medical Center, Omaha, NE 68198 USA; 5 Broad Institute of MIT and Harvard, Cambridge, MA 02142 USA; 6 The ChemoGenix CRISPR Screening Platform of the Institute for Research in Immunology and Cancer (IRIC), Université de Montréal, Montréal, QC H3T 1J4 Canada

**Keywords:** Targeted protein degradation, PROTAC, Heterobifunctional compound, Viability screen, MAP3K1, CRBN

## Abstract

Heterobifunctional compounds (HBCs) that facilitate target protein degradation (TPD) by recruiting an E3 ligase complex have emerged as therapeutic options for cancer. Current screening approaches often prioritize protein degradation over anticancer activity, potentially missing therapeutically relevant compounds. To address this, we developed a viability-based screening strategy using isogenic wild-type, *MAP3K1*-knockout, and *CRBN*-knockout cell lines to identify HBCs with target- and E3-ligase-dependent anticancer effects. This approach identified HBC-8, which is > 30-fold more potent than the parent MAP3K1 inhibitor and induces S-phase arrest, growth inhibition, and apoptosis in a MAP3K1- and CRBN-dependent manner. HBC-8 degrades MAP3K1; however, proteome-wide, genome-wide, and large-scale viability-based screens indicate that the anticancer activity of HBC-8 also relies on the disruption of the ubiquitin–proteasome system and protein translation machinery, extending beyond MAP3K1 degradation alone. Target dependence of HBC-8 was established using a large-scale viability screen in 869 cancer cell lines, and the anticancer effects correlated with the expression levels of the target (MAP3K1) and E3 ligase (CRBN). Furthermore, HBC-8 showed antitumor effects in an in vivo orthotopic ovarian cancer model without observable toxicity, supporting the use of isogenic cell lines for chemical genetic screens as a rapid, holistic approach to identify therapeutically relevant HBCs that also degrade the target protein.

**Supplementary Information:**

The online version contains supplementary material available at https://doi.org/10.1186/s43556-026-00632-z.

## Introduction

Heterobifunctional compounds (HBCs) represent a promising therapeutic modality that harnesses the cellular ubiquitin–proteasome system to degrade disease-relevant proteins [1–4]. The first rationally designed heterobifunctional compound (HBC), Protac1, was reported nearly 25 years ago [5]. Subsequent discovery and development of small molecules that bind to E3 ligase substrate receptors such as cereblon (CRBN) [6, 7] resulted in the rapid discovery of HBCs targeting diverse proteins of interest (POIs) [4, 8, 9] with several compounds advanced through to clinical trials [1, 2].

**Fig. 1:**
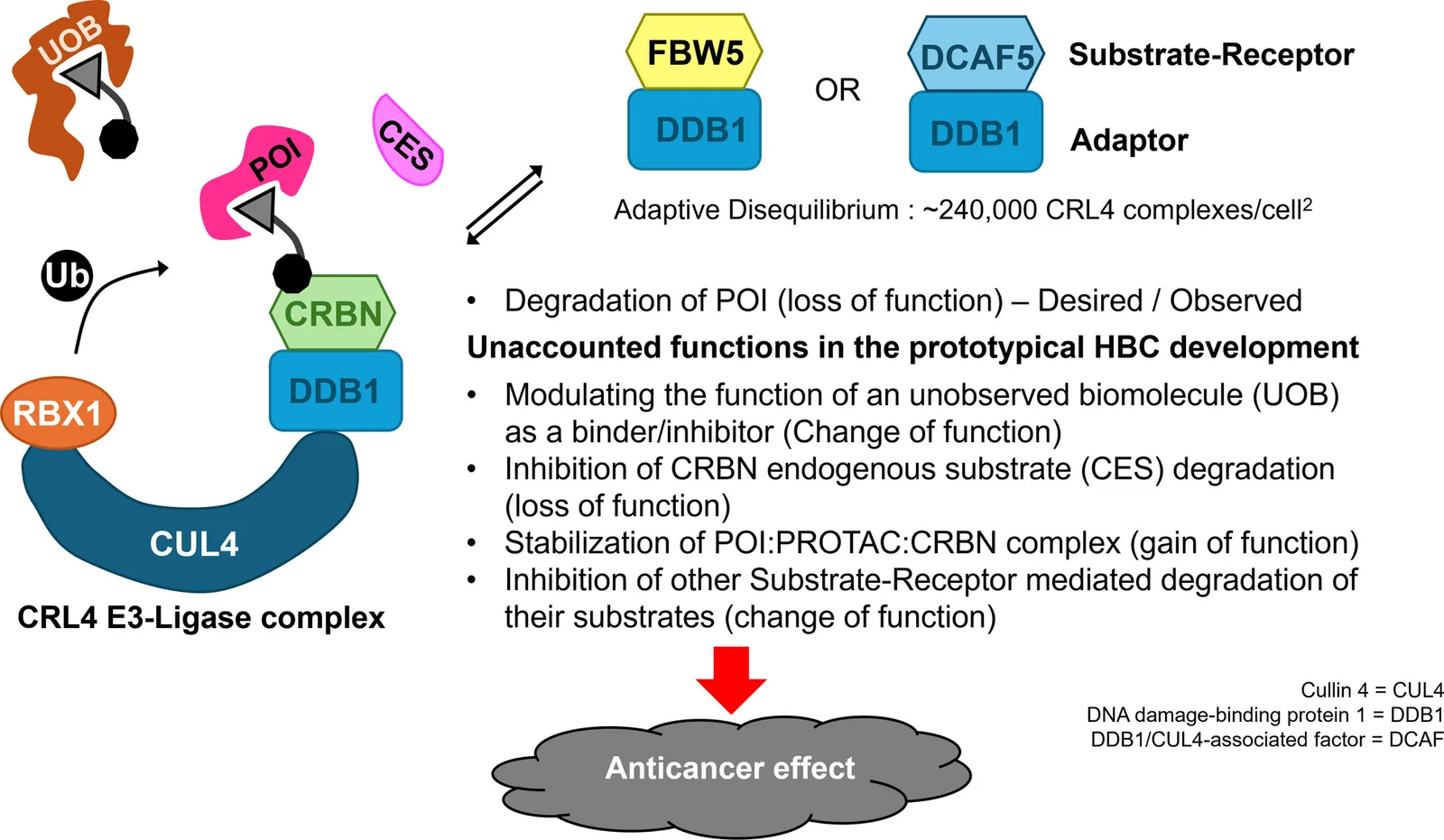
An overview of the various functions of HBC degraders that use CRBN targeting ligands

Current HBC discovery pipelines start with conjugating a POI-binding molecule to an E3-ligase ligand through linkers of varying length, rigidity, and composition. The resulting HBC library is screened in assays that report on POI degradation and/or ternary complex formation (POI::HBC::E3-ligase), and the hits identified from the screen are subjected to a series of competition assays to establish the mechanism of action (MOA). Despite significant progress, current approaches face significant limitations at various steps [1, 4] with screening approaches remaining understudied [10–12]. HBCs, due to their large molecular weights and large exposed polar surfaces, suffer from poor cellular permeability [13]. Yet, many primary screens utilize a cell-free system that cannot assess cellular permeability. In contrast, cell-based screens typically use endpoint degradation readout without accounting for biological processes and mechanistic insights [12, 14]. A lack of holistic consideration of all possible effects of HBCs (Fig. 1) overlooks the multifaceted nature of HBC mechanisms, potentially missing compounds with modest POI degradation but significant biological activity through alternative mechanisms that depend on the presence of POI and E3 Ligase.

HBCs are multi-functional molecules whose therapeutic effects extend beyond primary POI degradation. CRBN-based HBCs degrade neo-substrates beyond the intended target, posing significant biological effects [15–18]. The binding of an E3 ligase to the POI can cause second-degree degradation of POI partners, such as the secondary degradation of cyclin K through CDK12 degraders [19–21]. HBC interactions with E3 ligases significantly alter cellular ubiquitination dynamics. For example, CRBN-based HBCs recruit the CUL4 E3 ligase complex, and it is estimated that approximately 240,000 CUL4 complexes per cell could form through a rapid substrate-receptor exchange [22]. Even minor perturbations to this mechanism can therefore have profound substrate rewiring and cellular consequences. HBCs can also modulate the target protein function by altering its conformation, ability to interact with other proteins, and catalytic activity [22]. Recent work has also shown that DCAF substrate receptors compete for CUL4 binding, and modulating this competition, for example, by targeting DCAF5 in *SMARCB1*-mutant tumors, has therapeutic consequences [23, 24], thus demonstrating that HBC engagement of CUL4 machinery could influence endogenous pathways beyond primary POI degradation. These unaccounted functions contribute to HBC efficacy even when quantitative degradation appears suboptimal and complicate the structure–activity relationship (SAR). Therefore, it is crucial to expand evaluation criteria beyond POI degradation. To this end, we present a proof-of-concept study that used a viability-based screening strategy as a rapid and holistic approach to identify therapeutically relevant HBCs that also degrade MAP3K1.

Mitogen-activated protein kinase kinase kinase 1 (MAP3K1) is a critical regulator of MAPK and NF-κB pathways [25–30], and modulates cellular processes that contribute to cancer cell proliferation, migration, and apoptosis [31, 32]. Dysregulation of MAP3K1 signaling contributes to poor prognosis and disease progression across multiple tumor types, including breast, pancreas, and ovarian cancers [33–38]. Our laboratory discovered several quinoxaline analogs that exhibit anticancer activities [9, 39–45]. More recently, we showed quinoxaline urea analogs (such as IKAM1 and 51–106) selectively target MAP3K1 [9, 45, 46]. However, like many kinase inhibitors, these compounds require micromolar concentrations to inhibit cancer cell growth. Several studies have demonstrated improved anticancer efficacy of protein degraders compared to the corresponding inhibitors across different protein targets [47, 48]. Based on this, we hypothesized that protein degradation strategies could achieve enhanced potency.

To test this, we aimed to develop HBCs using IKAM-1 and/or 51–106 as MAP3K1-targeting warheads and evaluate them for anticancer activity that is dependent on MAP3K1 and CRBN, which addresses a limitation of current HBC screening approaches. A viability-based screen using isogenic wild-type, *CRBN* KO, and *MAP3K1* KO cell lines simultaneously assessed cellular permeability, target dependence, and anticancer activity under physiologically relevant conditions. The screen identified HBC-8, a quinoxaline-pomalidomide conjugate, whose anticancer activity depends on the presence of both MAP3K1 and CRBN. Consistent with reported studies with other systems, HBC-8 was > 30-fold more potent than the parent inhibitor 51–106 (iMAP3K1). Through comprehensive mechanistic characterization, including large-scale cell line profiling, proteome-wide analysis, and genome-wide CRISPR screening, we show that HBC-8 operates through mechanisms that extend beyond MAP3K1 degradation. This work provides a practical alternative approach to rapidly identify therapeutically relevant HBCs whose anticancer activity depends on the presence of the targeted proteins.

## Results

### A chemical genetic screening identifies HBC-8 with MAP3K1- and CRBN-dependent growth inhibitory activity

**Fig. 2:**
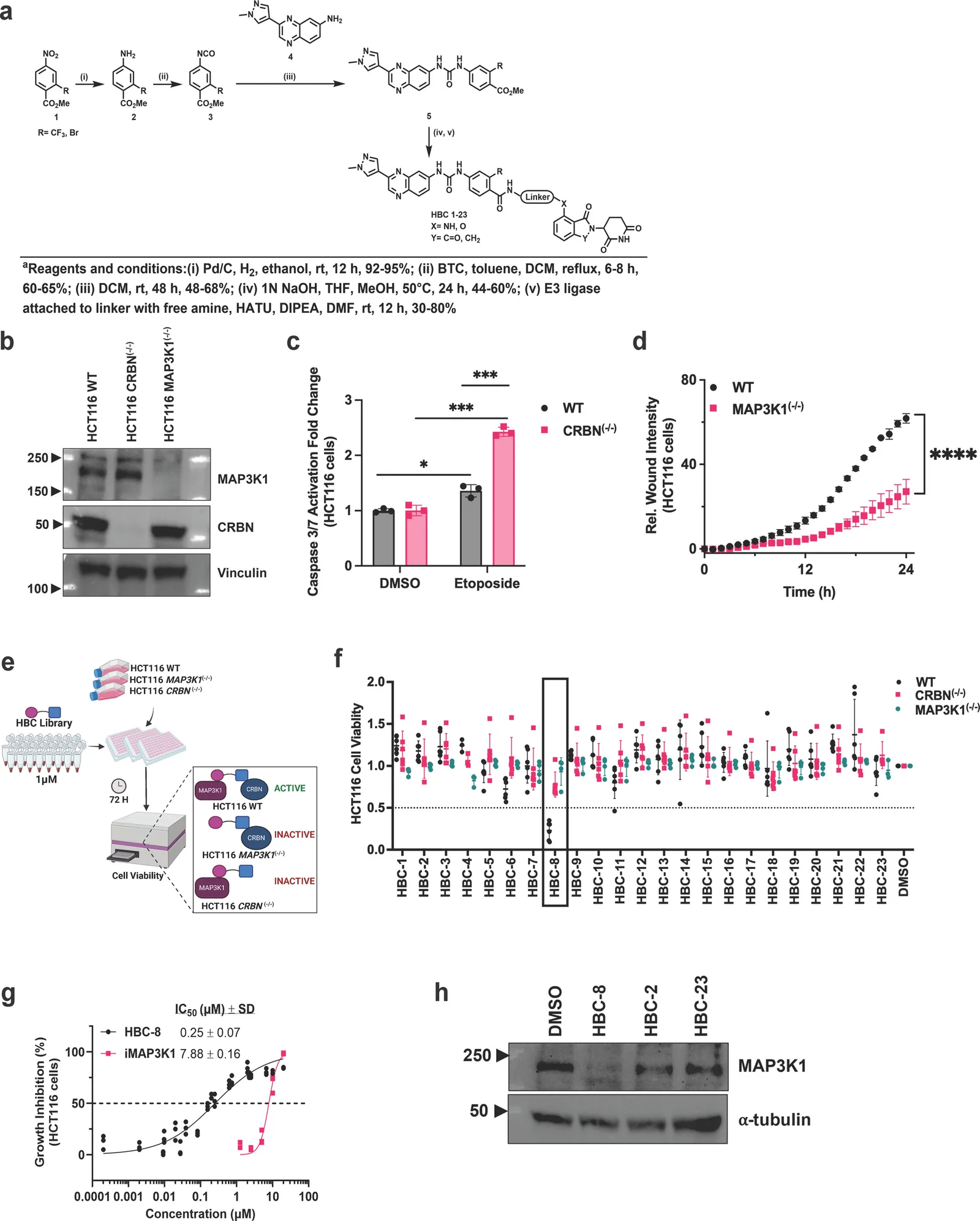
Discovery of MAP3K1-targeted HBC: **a** General schematic that describes the synthesis of MAP3K1-targeted HBC. **b** Immunoblot validation of HCT116 wild type (WT), HCT116 *CRBN* KO (*CRBN*^*−/−*^), and HCT116 *MAP3K1* KO (*MAP3K1*^*−/−*^). Representative blot from (*n* = 3) biological replicates. Vinculin serves as a loading control. **c** Caspase 3/7 activity assay in HCT116 WT and *CRBN*^*−/−*^ cells treated with etoposide (10 μM) or DMSO control for 24 h. Data represent the mean ± SEM (*n* = 3). Statistical analysis was performed by two-way ANOVA (*p < 0.05, ***p < 0.001). **d** Wound healing assay comparing the migration capacity of HCT116 WT and *MAP3K1*^*−/−*^ cells over 24 h. Relative wound intensity was measured at 1-h intervals to assess wound closure rates. Data represent mean ± SEM (*n* = 3). Statistical analysis was performed by two-way ANOVA (****p < 0.0001). **e** Workflow of viability-based phenotypic screening strategy to identify HBC compounds active in a MAP3K1- and CRBN-dependent manner, showing predicted mechanistic outcome. *Created in BioRender. Vishwakarma, K. (2026) *https://BioRender.com/owisvaw. **f** Viability-based phenotypic screening of HBC library in isogenic HCT116 cell lines. HCT116 WT (black), *CRBN*^*−/−*^ (magenta), and *MAP3K1*^*−/−*^ (teal) cells were treated with 1 μM of each quinoxaline-based HBC compound (HBC-1–23) for 72 h. We assessed cell viability and identified HBC-8 (boxed) as the lead compound, showing selective growth inhibition in WT cells but not in either knockout cell line. Data are presented as mean ± SEM (*n* = 6 biological replicates per condition). Statistical analysis for each compound is reported in Table S2. **g** Growth inhibition assay in HCT116 WT cells treated with varying concentrations of HBC-8 (magenta) and iMAP3K1 (black) for 72 h. Data are presented as mean ± SEM; HBC-8, *n* = 6; iMAP3K1, *n* = 3 biological replicates. Statistical comparison of IC_50_ values by unpaired two-tailed t-test (*p* < 0.0001). **h** Western blot analysis of MAP3K1 protein levels in HCT116 WT cells treated with DMSO vehicle control or HBC compounds (HBC-2, HBC-8, and HBC-23) at 1 μM concentration for 24 h. α-tubulin serves as a loading control. Representative blot from (*n* = 3) biological replicates

To improve the potency of the quinoxaline-based MAP3K1 inhibitors, we used IKAM-1 and iMAP3K1 as the warheads, and the solvent-exposed region was used as the exit vector to conjugate linkers [9, 46]. We synthesized a library of 23 heterobifunctional compounds (HBCs) that differ in linker length, linker composition, and warheads (Fig. 2a and Table S1). To systematically screen the HBC library, we developed a single assay system that simultaneously evaluates cell permeability, target requirement, and anticancer activity, using isogenic HCT116 wild-type (WT), *CRBN*-knockout (*CRBN *KO), and *MAP3K1*-knockout (*MAP3K1 *KO) cell lines. The absence of MAP3K1 and CRBN in the knockouts was confirmed by immunoblot (Fig. 2b). CRBN has been reported to confer resistance to etoposide-induced apoptosis [49]. Consistently, in *CRBN* KO cells treated with etoposide, increased caspase activation relative to WT cells was observed (Fig. 2c). Similarly, as MAP3K1 regulates c-JUN-mediated cell migration [50]. *MAP3K1* KO cells showed significantly reduced migratory capacity in a wound-healing assay (Fig. 2d). These studies ensured that the cell line models used in our screening system could reliably detect compounds whose activity depends on the presence of both the target protein (MAP3K1) and the E3 ligase (CRBN).

The HBC library was screened for inhibition of cell growth in HCT116 WT, *CRBN *KO, and *MAP3K1 *KO cells (Fig. 2e). The goal was to identify HBCs that inhibited growth of WT but not KOs, which would indicate cell permeability and growth-inhibitory activity that is dependent on both MAP3K1 and CRBN. The screen identified HBC-8 as the only HBC that showed growth-inhibitory activity in HCT116 WT cells and, more importantly, did not exhibit anticancer activity in cell lines lacking either CRBN or MAP3K1 (Fig. 2f and Table S2). We screened the HBC library in two additional WT cancer cell lines, T47D (Fig. S1a) and MCF7 (Fig. S1b), and again, only HBC-8 exhibited anticancer activity. Consistent with reported studies that showed degraders are more potent than the corresponding inhibitors, HBC-8 was > 30-fold more potent than the iMAP3K1 (Fig. 2g). The stringent hit rate (1/23 compounds) along with the lack of activity in closely related analogs (HBC-2 and HBC-23) suggests that effective target protein and E3-ligase engagement require a precise molecular framework to form the MAP3K1::HBC-8::CRBN complex. HBC-2 differs from HBC-8 by a single-carbon-shorter linker, and HBC-23 by substitution of the warhead -CF_3_ with -Br. The high structural similarity of HBC-2 and HBC-23, as compared to HBC-8, makes them ideal negative controls. HBC-2 and HBC-23 failed to degrade MAP3K1 (Fig. 2h), indicating that both precise linker length and substitution pattern are required for target engagement.

### Genetic and chemical approaches show that HBC-8 activity requires both MAP3K1 and CRBN

**Fig. 3:**
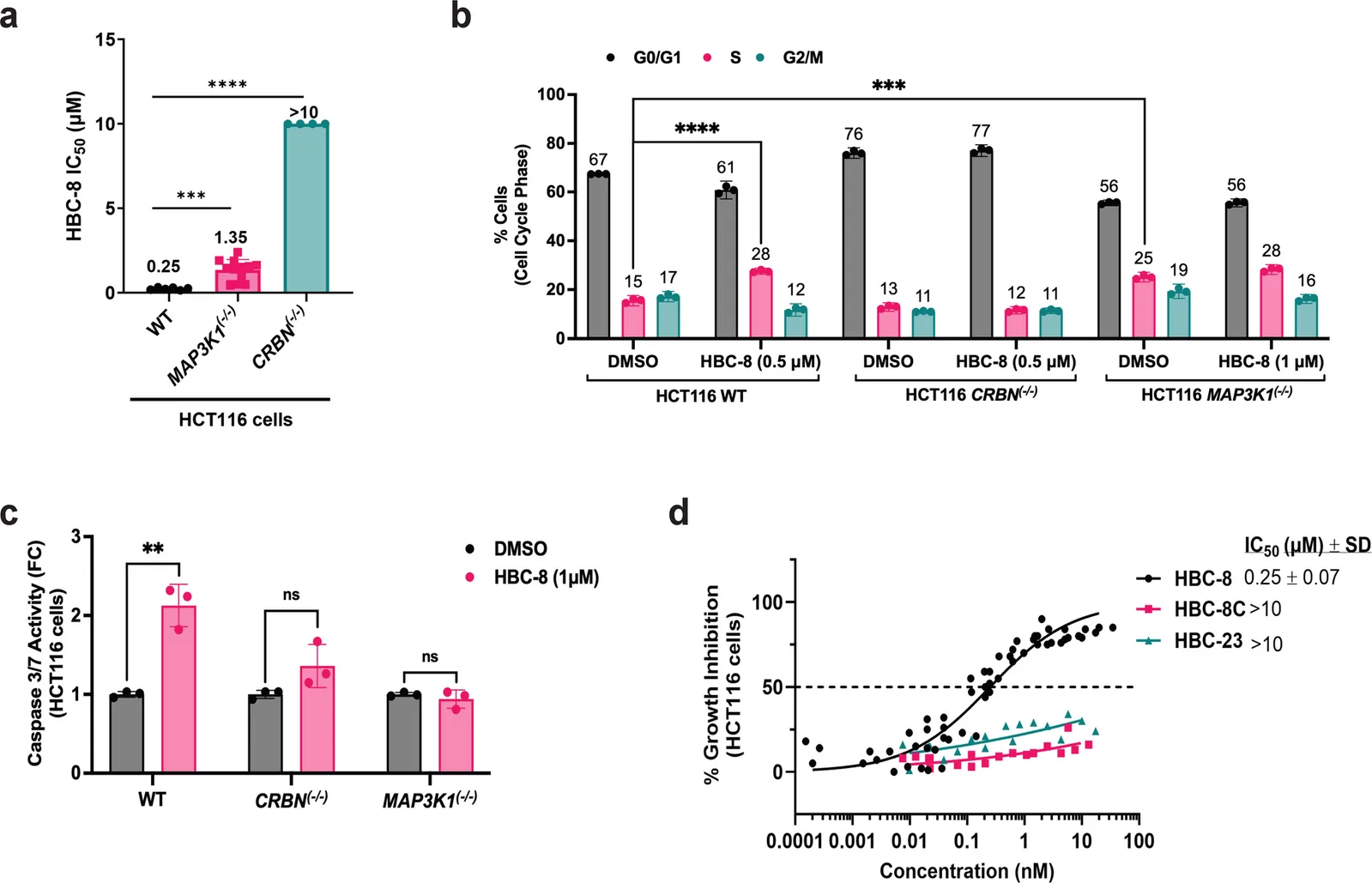
Genetic and chemical-based mechanism of action validation of HBC-8: **a** IC_50_ averages of HBC-8 in HCT116 WT (*n* = 6), *MAP3K1*^−/−^ (*n* = 12), and *CRBN*^−/−^ (*n* = 4) from cell viability assays in cells treated with varying concentrations of HBC-8 for 72 h. Data are presented as mean ± SEM. Statistical analysis was performed using an unpaired two-tailed t-test with Welch’s correction (***p < 0.001, ****p < 0.0001). **b** Cell cycle analysis in HCT116 WT, *CRBN*^−/−^, and *MAP3K1*^−/−^. Cells were treated with DMSO or HBC-8 for 24 h. WT and *CRBN*^−/−^ cells were treated with 0.5 μM HBC-8; *MAP3K1*^−/−^ cells were treated with 1 μM HBC-8, followed by cell cycle analysis. Numbers indicate the percentage of cells in each phase. Data are presented as mean ± SEM (*n* = 3 biological replicates). Statistical analysis was performed using unpaired t-tests (***p < 0.001, ****p < 0.0001). **c** Caspase 3/7 activity in HCT116 WT, *CRBN*^−/−^, and *MAP3K1*^−/−^. Cells were treated with DMSO or HBC-8 (1 μM) for 24 h. Caspase 3/7 activity was measured using the Caspase-Glo® 3/7 Assay System and normalized to cell viability measured by Alamar Blue. Data are presented as fold change (FC) relative to DMSO control, mean ± SEM, *n* = 3 biological replicates. Statistical analysis was performed using an unpaired t-test (**p < 0.01; ns, not significant). **d** Growth inhibition assay in HCT116 WT cells treated with varying concentrations of HBC-8, HBC-8C, and HBC-23 for 72 h. Data are presented as mean ± SEM; HBC-8, *n* = 6; HBC-8C and HBC-23, *n* = 3 biological replicates per condition

To confirm that HBC-8 activity depends on MAP3K1 and CRBN, we performed comprehensive genetic and chemical validation. The half-maximal inhibitory concentration (IC_50_) value of HBC-8 in WT cells is > 5-fold and > 20-fold lower than the IC_50_ value in *MAP3K1* KO and *CRBN* KO cells, respectively (Fig. 3a). To investigate the mechanism underlying the observed anti-proliferative effect, we examined cell cycle distribution across the isogenic HCT116 cell lines (Fig. 3b) and HeLa cells (Fig. S2a). Cell cycle analysis showed that HBC-8 induced S-phase arrest in WT cells, comparable to S-phase levels in DMSO-treated *MAP3K1 *KO cells, suggesting that pharmacological targeting of MAP3K1 phenocopies genetic loss of *MAP3K1*. HBC-8 did not affect the cell cycle of either *MAP3K1* KO or *CRBN* KO cells. We next tested whether HBC-8 also displays cytotoxicity by examining caspase activity, a marker of apoptosis induction. Caspase activity analysis showed that HBC-8 induced modest caspase 3/7 activation in WT but not in knockout cells (Fig. 3c). To validate the mechanism of HBC-8 chemically, we employed two negative control compounds: HBC-23 and N-methylated thalidomide, disrupting CRBN binding, HBC-8C [51]. HBC-8 showed dose-dependent antiproliferative activity in HCT116 cells (Fig. 3d) and in patient-derived organoids (Fig. S2b), while the control compounds were largely inactive. Similarly, only HBC-8, not the negative controls, affected the cell cycle (Fig. S2c) and induced caspase 3/7 activity (Fig. S2d). Together, these orthogonal chemical and genetic approaches show that HBC-8 activity depends on CRBN and MAP3K1, confirming its heterobifunctional mechanism.

### Large-scale cell line profiling identifies HBC-8 as a potent pan-cancer growth inhibitor with CRBN- and MAP3K1-dependent sensitivity

**Fig. 4:**
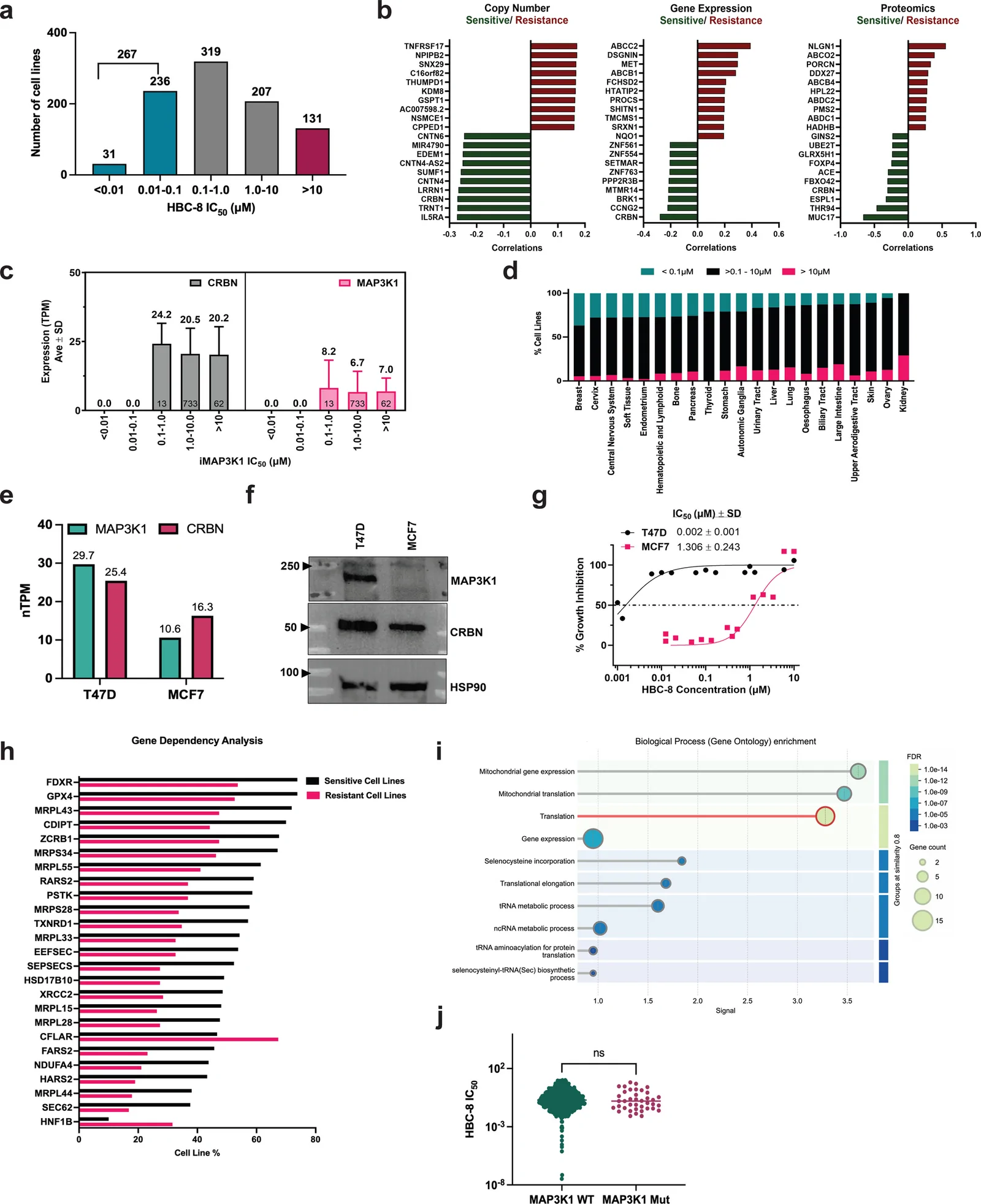
Large-scale cell line profiling of HBC-8: **a** Histogram of IC_50_ values across 869 cell lines: more than 260 cell lines (teal) exhibit HBC-8 IC_50_ values < 0.1 μM, whereas 131 cell lines (maroon) have IC_50_ values > 10 μM. **b** Top 10 significant DepMap copy number, gene expression, and proteomics biomarkers (q values < 0.05) of sensitivity to HBC-8 (log2(AUC)). **c** Cell lines from the PRISM screen were stratified by HBC-8 IC_50_ values and analyzed for *MAP3K1* (pink) and *CRBN* (black) mRNA expression levels from DepMap data. Expression values are shown as transcripts per million (TPM) ± standard deviation. Numbers at the base of each bar indicate the number of cell lines in each IC_50_ category. **d** Distribution of cell line sensitivity to HBC-8 across 21 cancer types with ≥ 10 cell lines screened in the PRISM analysis. IC_50_ values categorize cell lines as highly sensitive (< 0.1 μM, teal), moderately sensitive (> 0.1 and < 10 μM, black), or resistant (> 10 μM, pink). Data are presented as a percentage of total cell lines within each cancer type. **e** mRNA expression nTPM values of *MAP3K1* (teal bars) and *CRBN* (magenta bars) in T47D and MCF7 breast cancer cell lines. **f** Western blot analysis of MAP3K1 and CRBN protein levels in T47D and MCF7 cells at baseline. HSP90 serves as a loading control (*n* = 1). Molecular weight markers (kDa) are indicated on the left. **g** Growth inhibition assay in T47D and MCF7 cells treated with varying concentrations of HBC-8, for 120 and 72 h, respectively. Data are presented as mean ± SEM (*n* = 3 biological replicates). **h** Gene dependency analysis using DepMap CRISPR data (Chronos scores) comparing HBC-8-sensitive (*n* = 210, black bars) and resistant (*n* = 95, magenta bars) cell lines. **i** Gene Ontology Biological Process enrichment analysis of 19 genes showing differential dependencies in sensitive cell lines. **j** Comparison of HBC-8 IC_50_ values between cancer cell lines harboring wild-type *MAP3K1 *(*n* = 697) and *MAP3K1* mutations (*n* = 39) from the PRISM screen dataset (cell line mutational status was identified from DepMap). Each point represents an individual cell line’s sensitivity to HBC-8 treatment. No significant difference in drug sensitivity was observed between *MAP3K1* wild-type and mutant cell lines (Mann–Whitney test, *p* = 0.3224)

Next, we performed a colony formation assay using CD34 + white blood cell (WBC) precursors to assess HBC-8’s effect on normal hematopoiesis as a surrogate for toxicity. HBC-8 at a concentration of ≤ 0.1 μM did not affect normal colony formation (Fig. S3a). This suggests that cancers sensitive to low-nM levels of HBC-8 will have a favorable therapeutic window. Next, we examined the anticancer effects of HBC-8 in a large-scale PRISM (Profiling Relative Inhibition Simultaneously in Mixtures) profiling of 869 cancer cells across 28 cancer types (Fig. S3b). We identified 267 cell lines sensitive to HBC-8, with IC_50_ values of <0.1 μM, including 31 cell lines with high sensitivity (IC_50_ < 0.01 μM). In contrast, 131 cell lines were resistant, with IC_50_ values > 10 μM (Fig. 4a). Correlating HBC-8 sensitivity (area under the dose–response curve, AUC) with multi-omics features in the DepMap portal, gene expression, protein level, and copy number, identified CRBN as a driver of sensitivity across all three modalities. Additional sensitivity biomarkers included ubiquitin–proteasome components (UBE2T, FBXO42), whereas resistance was driven predominantly by drug efflux: multiple ABC transporters (ABCB1, ABCC2, ABCG2) correlated with reduced sensitivity, consistent with efflux limiting intracellular accumulation of a large, lipophilic degrader (Fig. 4b) [52, 53]. Sensitivity showed a weak but significant negative correlation with *CRBN *mRNA and a weak, non-significant trend with *MAP3K1 *mRNA (Figs. 4c, Fig. S3c). Further correlation analysis using shRNA, metabolomics, and repurposed compound datasets is summarized in Fig. S3d. Analysis of sensitivity patterns across 21 types of cancer showed that breast cancer had the highest proportion of sensitive cell lines (Fig. 4d). Two breast cancer lines T47D and MCF7 with differing endogenous expressions of MAP3K1 and CRBN (Fig. 4e, f), exhibited > 500-fold difference in sensitivity to HBC-8 (Fig. 4g). To identify baseline genetic dependencies distinguishing HBC-8–sensitive from –resistant cell lines, we compared their pre-existing DepMap CRISPR dependency profiles, independent of HBC-8 treatment (Fig. 4h). Of 25 genes differing significantly in dependency, 19 were more essential in sensitive lines and were enriched for cytoplasmic and mitochondrial translation machinery (Fig. 4i). Among the cell lines with WT or mutated *MAP3K1*, HBC-8 effectively inhibited cell growth regardless of *MAP3K1* mutation status (Fig. 4j), which is consistent with degrader characteristics [54, 55].

### HBC-8 induces its effects through ternary complex-mediated proteasomal degradation of MAP3K1 and perturbs corresponding downstream signaling

**Fig. 5:**
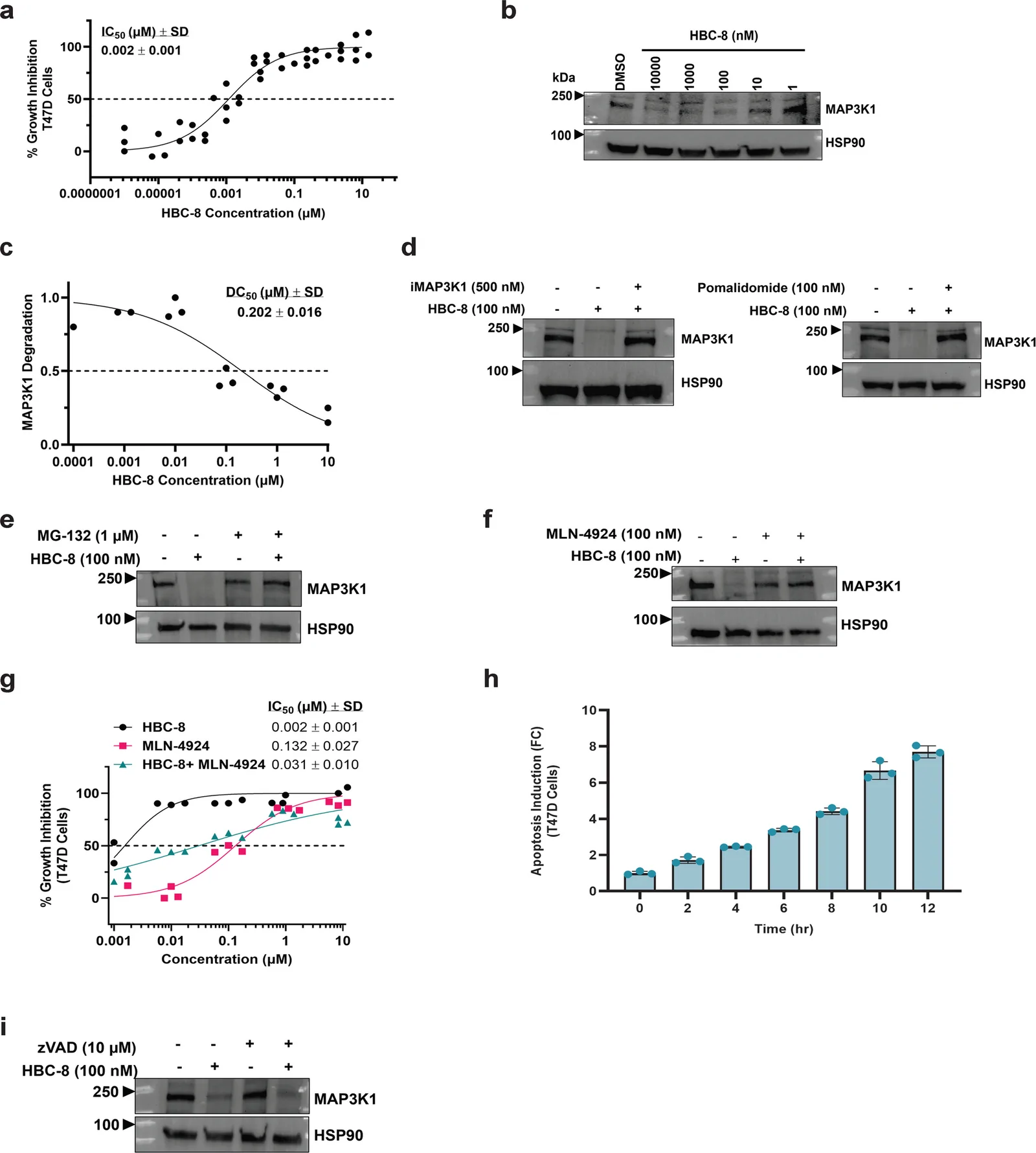
Mechanism of HBC-8-induced MAP3K1 degradation: **a** Growth inhibition assay in T47D cells treated with varying concentrations of HBC-8 for 120 h. Data presented as mean ± SEM, *n* = 3 biological replicates. **b** Western blot analysis showing MAP3K1 protein levels in T47D cells following 12-h treatment with varying concentrations of HBC-8 or DMSO vehicle control. **c** DC_50_ curve using MAP3K1 protein levels from (B), quantified by ImageJ, and values are normalized to the DMSO control. Data points represent individual measurements with a fitted curve with a DC_50_ of 0.2 μM. **d** Western blot analysis of T47D cells co-treated with HBC-8 (100 nM) and iMAP3K1 (500 nM) or Pomalidomide (100 nM) for 12 h. **e** Western blot analysis of T47D cells co-treated with HBC-8 (100 nM) and/or MG-132 (1 μM) for 12 h. **f** Western blot analysis of T47D cells co-treated with HBC-8 (100 nM) and/or MLN-4924 (100 nM) for 12 h. **g** Growth inhibition assay in T47D cells treated with HBC-8, MLN-4924, or combination treatment for 120 h. Cells were treated with varying concentrations of HBC-8 alone, MLN-4924 alone, or HBC-8 + MLN-4924 (1:1 ratio). Data are presented as mean ± SEM (*n* = 3 biological replicates). **h** Time-course analysis of apoptosis induction in T47D cells treated with HBC-8 (100 nM). Data presented as fold change in apoptosis induction (green fluorescence/phase contrast ratio) relative to baseline at indicated time points. Data are presented as mean ± SEM (*n* = 3 biological replicates). **i** Western blot analysis of T47D cells co-treated with HBC-8 (100 nM) and/or zVAD (10 μM) for 12 h. For each western blot, a representative blot from (*n* = 3) biological replicates is shown. HSP90 serves as a loading control; molecular weight markers (kDa) are indicated on the left

To investigate the molecular mechanism underlying the anticancer activity of HBC-8, we examined MAP3K1 degradation in sensitive cell lines: T47D, luminal B breast cancer (IC_50_ ~ 2 nM) (Fig. 5a), and NCIH1048, small cell lung cancer (IC_50_ ~ 0.2 nM) (Fig. S4a). HBC-8 induced dose-dependent degradation of MAP3K1 (Fig. 5b, Fig. S4b), with a half-maximal degradation (DC_50_) of 200 nM and 45 nM, in T47D and NCIH1048, respectively (Fig. 5c, Fig. S4c). Competition with excess iMAP3K1 and pomalidomide blocked HBC-8-mediated MAP3K1 degradation (Fig. 5d, Fig. S4d). Similarly, co-treatment of HBC-8 with a proteasome inhibitor, MG-132, rescued HBC-8-mediated MAP3K1 degradation (Fig. 5e, Fig. S4e). The formation of a stable ternary complex, *i.e.*, MAP3K1::HBC-8::CRBN-CUL4-RBX1, depends on neddylation of CUL4, which prevents substrate receptor complex exchange [22, 56–58]. Co-treatment of HBC-8 with the neddylation inhibitor MLN-4924 prevented MAP3K1 degradation (Fig. 5f, Fig. S4f) and increased the IC_50_ by > 10-fold in T47D and > 50-fold in HeLa cells (Fig. 5g, Fig. S4g). HBC-8 induced cellular apoptosis as evidenced by cleavage of PARP in the NCIH1048 (Fig. S4h) and a time-dependent increase in cellular apoptosis monitored by live cell imaging over 12 and 24 h (Fig. 5h, Fig. S4i). MAP3K1 has a well-defined caspase 3 cleavage site [59] and caspase activation drives widespread, non-selective cleavage of numerous cellular proteins [60]; MAP3K1 loss could therefore represent caspase-mediated cleavage rather than targeted degradation. To distinguish these possibilities, we co-treated cells with HBC-8 and the pan-caspase inhibitor Z-VAD-FMK (zVAD) and monitored MAP3K1 (Fig. 5i, Fig. S4j). MAP3K1 loss persisted under caspase inhibition; together with the MG-132 and MLN-4924 rescue data, this supports that HBC-8-mediated MAP3K1 degradation occurs through the ubiquitin–proteasome system.

MAP3K1 regulates various signaling pathways, including JNK/cJUN, MEK/ERK, and NF-κB (Fig. S5a). To assess the effect of HBC-8 on MAP3K1-associated signaling, we performed immunoblot analyses of treated cells, which showed reduced p-IKKβ and p-ERK with a concurrent increase in p-JNK (Fig. S5b). Increased p-JNK resulted from reduced p-ERK-mediated DUSP4 activity, a critical JNK phosphatase signaling [61]. Sustained JNK activation induces apoptosis through mitochondrial and AP-1-mediated pathways [59, 62, 63], likely contributing to HBC-8’s cytotoxicity, while reduced p-ERK underlies cytostatic effects. Immunoblot analysis of > 20 kinases and the members of the CUL4 complex revealed that HBC-8 is a selective MAP3K1 degrader (Fig. S5c, S5d). Among these, CDK4 levels displayed a slight reduction, whereas CDK1, CDK2, and other CDKs were unchanged. This selective reduction of CDK4, a G1/S regulator, could be attributed to the cell-cycle arrest induced by HBC-8 (Fig. 3b). Consistent with published data, our Immunoprecipitation–Immunoblotting (IP-IB) analysis showed that MAP3K1 complexes with the CUL4A-DDB1-DCAF7 [64] and reports, for the first time, a baseline direct or indirect MAP3K1-CRBN interaction (Fig. S5e). These mechanistic results indicate that HBC-8 degrades MAP3K1 through a heterobifunctional mechanism requiring ternary complex formation and proteasome-mediated degradation, and that it perturbs MAP3K1 signaling networks.

### Proteome-wide profiling reveals that HBC-8 alters protein homeostasis with a distinct pattern when compared to either MAP3K1 or CRBN loss

**Fig. 6:**
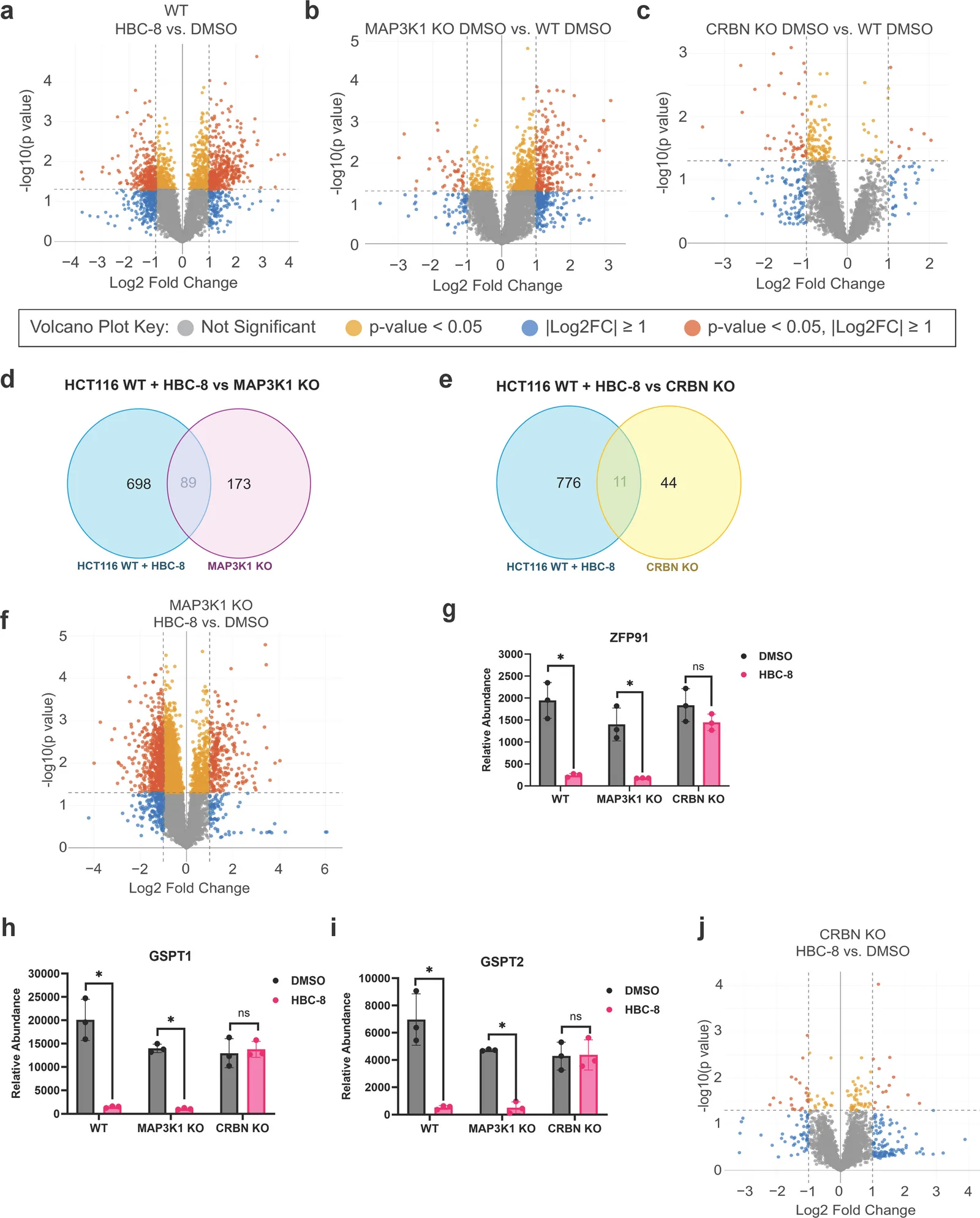
Proteome-wide profiling of HBC-8: volcano plots showing differentially expressed proteins (fold change ≥ 2, *p* < 0.05) in HCT116 cells: **a** WT + HBC-8 (0.5 μM) vs WT + DMSO, 72 h; **b** *MAP3K1* KO + DMSO vs WT + DMSO; **c** *CRBN* KO + DMSO vs WT + DMSO. Venn diagrams showing the overlap of HBC-8-altered proteins between **d** HBC-8-treated WT cells and *MAP3K1* KO baseline, and **e** HBC-8-treated WT cells and *CRBN* KO baseline. **f** Volcano plots of differentially expressed proteins (fold change ≥ 2, *p* < 0.05) in *MAP3K1* KO + HBC-8 (2 μM) vs *MAP3K1* KO + DMSO. Relative abundance of the top proteins downregulated after HBC-8 treatment: **g** ZFP91, **h** GSPT1, **i** GSPT2. **j** Volcano plots of differentially expressed proteins (fold change ≥ 2, *p* < 0.05) in *CRBN* KO + HBC-8 (10 μM) vs *CRBN* KO + DMSO. *n* = 3 biological replicates

To further investigate the mechanism underlying HBC-8’s antiproliferative activity, we conducted proteome-wide profiling of cells treated with HBC-8. We performed comparative proteomic profiling of HCT116 WT and KO cells treated with HBC-8 (Fig. 6a-c). We summarize the significantly altered proteins (> 2-fold change with *p* < 0.05) in Table S3. Of the 787 proteins altered by HBC-8, only 89 (11.3%) overlapped with *MAP3K1* KO (Fig. 6d), and 11 (1.4%) with *CRBN* KO (Fig. 6e). These results suggest that > 85% of proteome-wide changes are unique to HBC-8 and the observed antiproliferative effects associated with HBC-8 are not exclusively due to the functional loss of MAP3K1 or CRBN. Similarly, GO analysis of differentially altered proteins in WT cells treated with HBC-8, *MAP3K1* KO, and *CRBN* KO cells showed distinct pathway signatures (Fig. S6a –c). To further delve into MAP3K1-independent effects, *MAP3K1* KO cells were treated with HBC-8. We identified 241 altered proteins independent of MAP3K1 presence (Fig. 6f, Fig. S6d), including well-characterized CRBN neo-substrates, GSPT1 [65, 66] and ZFP91 [67] (Fig. 6g-i). This suggests the CRBN binding moiety in HBC-8 can recruit CRBN neo-substrates. Conversely, *CRBN* KO cells treated with HBC-8 showed only 32 altered proteins (Fig. 6j, Fig. S6e), indicating asymmetric dependence on CRBN and highlighting the CRBN-CUL4 system’s role in HBC-8’s biological function beyond MAP3K1 degradation. GO analysis of these conditional treatments revealed that HBC-8 effects in *MAP3K1* KO cells involved mitotic processes, while effects in *CRBN* KO cells were limited to metabolic processes (Fig. S6f, g). Given MAP3K1’s E3 ligase activity, analysis of predicted MAP3K1 E3-ligase substrates [68] showed no significant accumulation in *MAP3K1* KO or HBC-8-treated WT cells (Fig. S6h, i), suggesting these are not direct substrates in HCT116 cells. Together, these findings suggest that HBC-8 alters protein homeostasis through mechanisms that extend beyond MAP3K1 degradation.

### CRISPR screening identifies ubiquitin–proteasome and translation machinery as a dependency of HBC-8

**Fig. 7:**
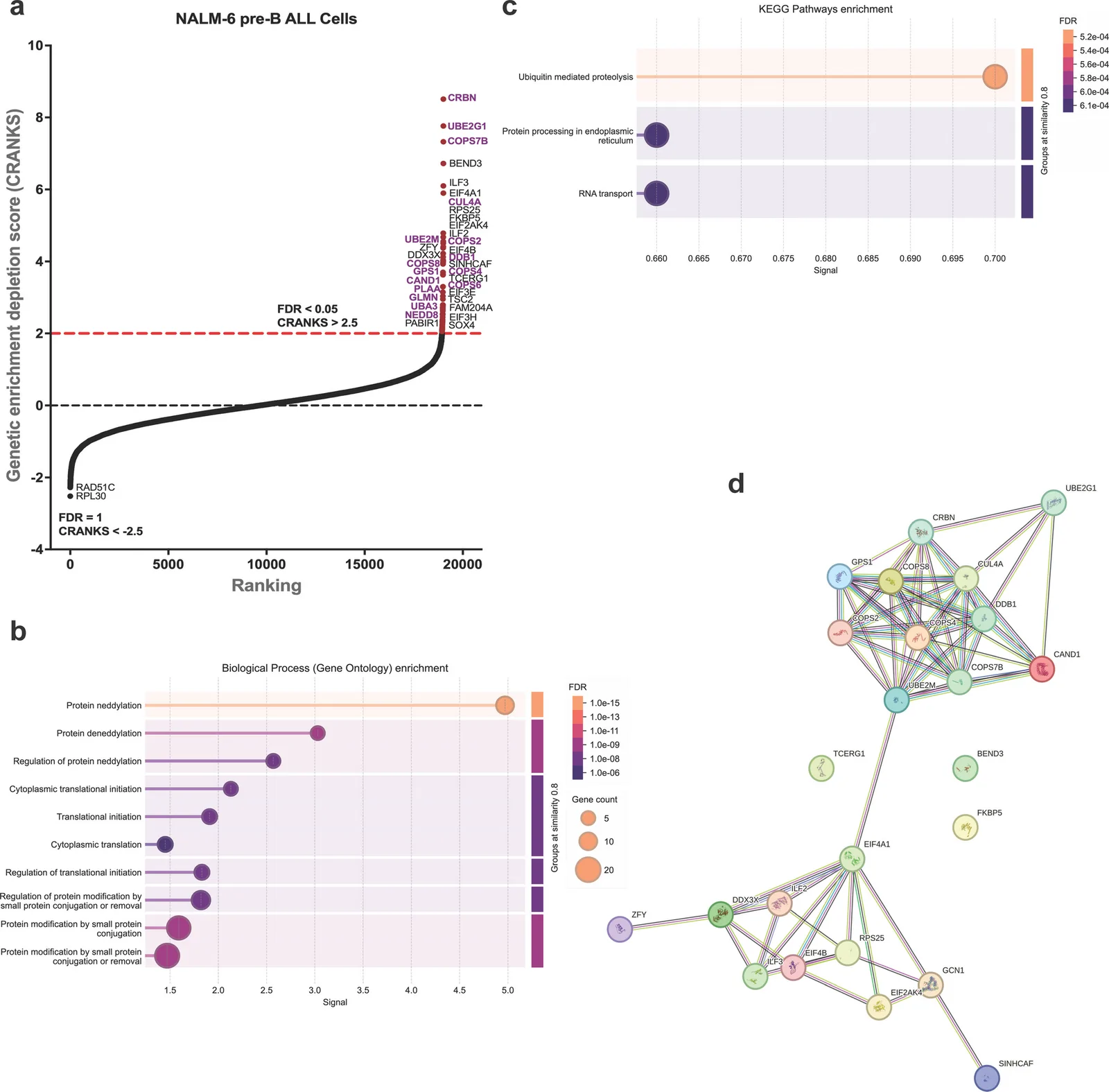
Genome-wide profiling of HBC-8: **a** Gene enrichment depletion score (CRANKS score) plotted against gene ranking for 19,085 genes screened. Genes with positive CRANKS scores above the significance threshold (red dashed line at CRANKS = 2.5) are top-scoring genes that confer resistance. **b** Gene Ontology biological process enrichment analysis of the top 25 genes (FDR < 0.001, CRANKS > 2) identified from the CRISPR screen. **c** KEGG pathway enrichment analysis of the rescue genes from CRISPR screening (FDR < 0.1). Gene Ontology and KEGG pathway enrichment analyses were performed using the STRING database; bubble size is proportional to the number of genes, and color intensity indicates statistical significance. **d** STRING database analysis showing known and predicted protein–protein interactions among the 25 genes identified in the CRISPR screen (CRANKS score > 2 and FDR < 0.001). (extended definition of nodes in Fig. S7c)

To systematically identify genetic determinants of HBC-8 sensitivity, we performed a genome-wide CRISPR knockout screen in NALM6 cells, a suspension cell line sensitive to HBC-8 (IC_50_ = 0.35 µM; Fig. S7a), with high, inducible Cas9 editing efficiency and an established genome-wide extended-knockout (EKO) library (IRIC ChemoGenix), making it a standard platform for unbiased dropout screens. NALM6 cells were treated with a concentration of HBC-8 that reduced population doubling ~ 5-fold relative to DMSO, chosen to identify gene knockouts that rescue cells from its growth-inhibitory effect. From > 19,000 genes screened, we identified 55 genes whose loss conferred resistance (Fig. 7a). *CRBN* emerged as the top resistance hit (CRANKS score > 8), and its E2 ubiquitin-conjugating partner *UBE2G1* ranked second with a CRANKS score of ~7.5. Other hits include ubiquitin–proteasome components (*CUL4A*, *CAND1*, *NEDD8*) and COP9 signalosome subunits (*COPS1*, *COPS2*, *COPS4*, *COPS7B*, *COPS8*), confirming dependence on intact protein degradation machinery. Translation initiation factors (*EIF4A1*, *EIF4B*, *EIF3E*, *EIF4G1*) also emerged as high-scoring hits, consistent with PRISM cell line profiling that showed mitochondrial ribosomal dependencies in sensitive cells. GO analysis revealed enrichment in protein neddylation, cytoplasmic translation initiation, and ubiquitin-dependent degradation (Fig. 7b). Kyoto Encyclopedia of Genes and Genomes (KEGG) analysis indicated enrichment in ubiquitin proteasomal machinery and protein translation pathways (Fig. 7c). Protein levels of dependency genes showed no significant changes in our proteomics dataset (Fig. S7b), suggesting these represent proteins necessary for the ubiquitin–proteasome machinery or downstream vulnerabilities rather than direct degradation targets. STRING network analysis using genes with FDR < 0.001 revealed two major clusters: ubiquitin–proteasome system centered on CRBN and translation initiation complex (Fig. 7d). Although MAP3K1 was not among the significant rescue genes, network analysis identified MAP3K1 interactors, FKBP5, DDB1, and COPS6 (Fig. S7c). FKBP5 regulates NF-κB signaling and stress response with MAP3K1 [25, 69]. COPS6 promotes MAP3K1 ubiquitination, stabilizing c-JUN [70]. DDB1 is associated with MAP3K1 in the CUL4 complex during DNA damage response [64]. These interactions suggest that MAP3K1 sits at the center of regulatory networks involving stress response, protein degradation, and DNA damage signaling, and that HBC-8 perturbs these crucial signaling networks as the mechanism underlying its therapeutic effects.

### HBC-8 inhibits ovarian tumor growth in vivo through cytostatic and cytotoxic mechanisms

**Fig. 8:**
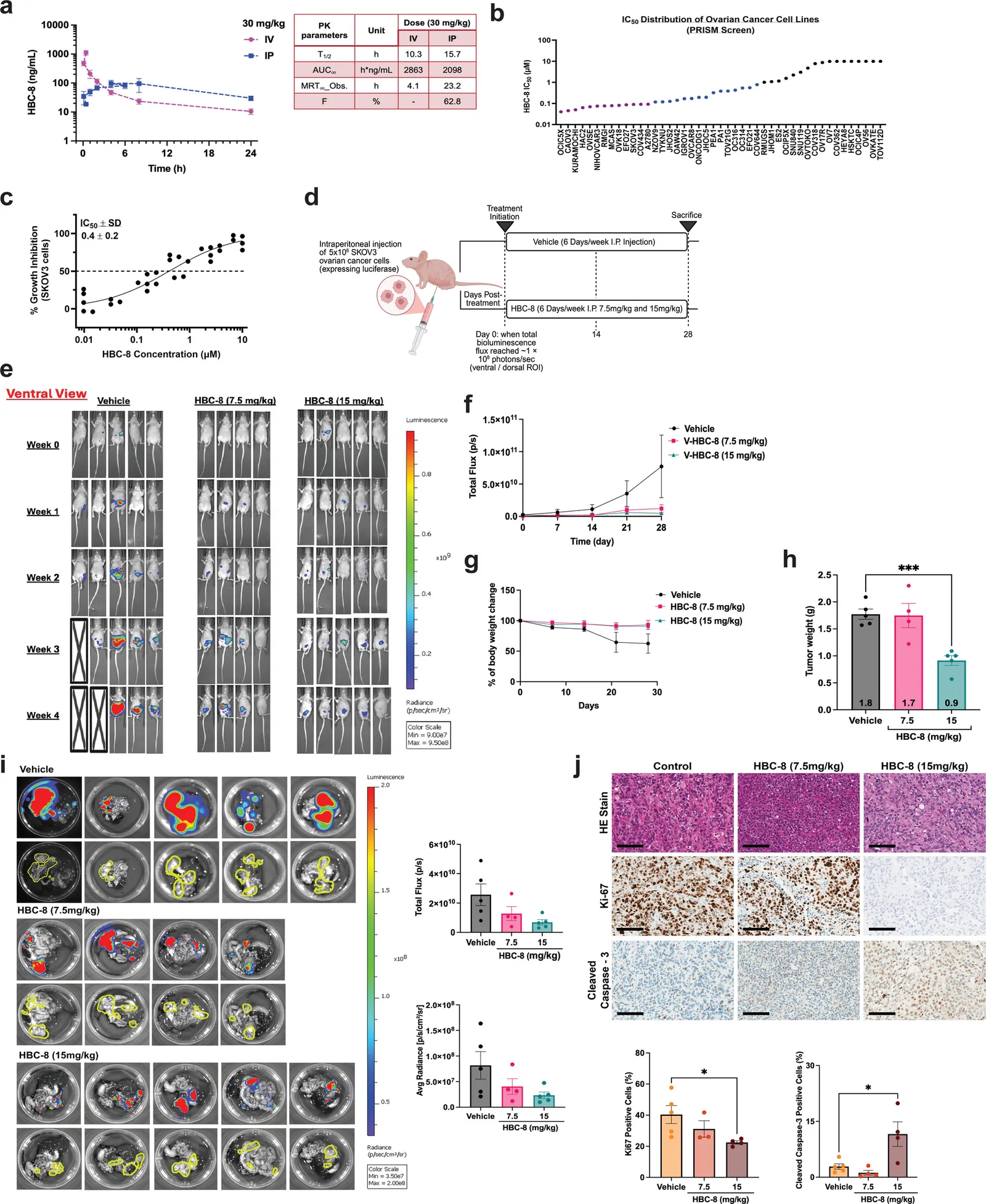
Evaluation of HBC-8 efficacy in SKOV3-luc intraperitoneal tumor model: **a** PK study (IPT and IV) data (*n* = 3). (Table) Summary of the PK parameters estimated by the non-compartmental model using WinNonlin 8.3. **b** IC_50_ values for HBC-8 across 45 ovarian cancer cell lines (from the PRISM viability screen), ranked by sensitivity: most sensitive (purple) to most resistant (black). Cell line names are displayed on the x-axis. Data represent IC_50_ values (y-axis). **c** Growth inhibition assay in SKOV3 ovarian cancer cells treated with varying concentrations of HBC-8 for 72 h. Data are presented as mean ± SEM (*n* = 3 biological replicates). **d** Schematic of the experimental workflow. SKOV3-luc cells (5 × 10⁶) were injected intraperitoneally into nude mice. *Created in BioRender. Vishwakarma, K. (2026) *https://BioRender.com/i3wi9zt. **e** Representative ventral IVIS images of mice treated with vehicle or HBC-8 (7.5 mg/kg or 15 mg/kg) at the indicated time points. **f** Quantification of total bioluminescent flux (photons/sec) over 28 days. Data are presented as mean ± SEM (vehicle, *n* = 5; HBC-8 7.5 mg/kg, *n* = 4; HBC-8 15 mg/kg, *n* = 5). **g** The percentage change in body weight in mice treated with vehicle or HBC-8 (7.5 mg/kg or 15 mg/kg) over a 28-day treatment period. Data are presented as mean ± SEM (vehicle, *n* = 5; HBC-8 7.5 mg/kg, *n* = 4; HBC-8 15 mg/kg, *n* = 5). **h** Tumor weight measurement of mice treated with vehicle or HBC-8 (7.5 mg/kg or 15 mg/kg). Data represent mean ± SEM. An unpaired two-tailed t-test was used to determine statistical significance; ***p = 0.0002. **i** Ex vivo (ventral) IVIS imaging of mice treated with vehicle or HBC-8 (7.5 mg/kg or 15 mg/kg). Data represents SEM (vehicle, *n* = 5; HBC-8 7.5 mg/kg, *n* = 4; HBC-8 15 mg/kg, *n* = 5). **j** Representative images of histological and immunohistochemical analysis of tumor tissues. H&E staining, Ki-67, and cleaved caspase-3 (CC3) in tumors from vehicle- and HBC-8–treated mice, with respective quantifications. Scale bar, 100 µm. Data represent mean ± SEM. An unpaired two-tailed t-test was used to determine statistical significance; *p < 0.05 (Extended images are reported in Fig. S8d)

We conducted a pharmacokinetic analysis to evaluate HBC-8’s therapeutic potential in vivo. HBC-8 administered at 30 mg/kg had favorable intraperitoneal (IPT) bioavailability (F ~ 62%) and an extended residence time (Fig. 8a). Therefore, we selected an ovarian cancer model to assess the in vivo efficacy of HBC-8. For the efficacy study, we selected two doses (7.5 and 15 mg/kg) expected to remain within a well-tolerated range while achieving exposures sufficient for anti-tumor activity. Ovarian cancer is known to metastasize to the peritoneal cavity rapidly, and IPT administration is therefore the optimal route [71–74]. The PRISM screen identified several ovarian cancer cell lines that were sensitive to HBC-8 (Fig. 8b). We selected the SKOV3 cell line based on its sensitivity profile and the availability of the SKOV3-luciferase cell line to establish the orthotopic model that we recently reported [75] (Fig. 8c). Mice bearing SKOV3-luciferase tumors were enrolled into treatment groups at a luminescent signal of 10^8^ photons/sec and received vehicle or HBC-8 (7.5 or 15 mg/kg) IPT for 4 weeks (Fig. 8d). Weekly IVIS imaging showed progressive tumor growth in the vehicle group, while treatment groups showed reduced signals (Fig. 8e, Fig. S8a). Analysis of the total flux showed that tumor burden progressively increased in the vehicle group, exceeding 1.2 × 10^11^ photons/sec at day 28. HBC-8 treatment reduced flux values in a dose-dependent manner (Fig. 8f, Fig. S8b). Some vehicle-treated animals lost up to 40% of their body weight. At the same time, HBC-8-treated mice maintained stable weights (Fig. 8g). The 15 mg/kg treated group showed a significant reduction in tumor weight (Fig. 8h, Fig. S8c), and ex vivo imaging confirmed dose-dependent tumor reduction (Fig. 8i). Immunohistochemistry showed that HBC-8 treatment reduced Ki-67-positive cells, a marker for cell proliferation, while increasing cleaved caspase-3 staining, a marker for induction of apoptosis (Fig. 8j, Fig. S8d). This confirms that in vivo anticancer effects were mediated by cytostatic and cytotoxic mechanisms.

## Discussion

The past decade has seen an exponential increase in reports that describe the discovery of heterobifunctional degraders that target cancer-associated proteins. Yet, a structure-guided approach to discovering heterobifunctional degraders remains elusive, leaving drug discovery programs to rely on synthesizing focused sets of degraders and screening them for activity. Current screening strategies prioritize target degradation and ternary-complex formation efficiency, often using recombinant protein systems that cannot capture cellular permeability or the intricate interplay between target engagement and therapeutic outcomes. Although degraders against various protein targets have been developed, this reductionist approach overlooks compounds with superior anticancer activity whose effects may arise from complex cellular network perturbations in addition to target loss. Beyond target degradation, several mechanisms that disrupt cellular homeostasis have been identified, and these unaccounted functions might contribute to the therapeutic effect of these compounds. The growing understanding of the complex mechanisms of action associated with HBCs warrants a holistic approach to screening and characterizing HBC hits.

To address these limitations, we developed a phenotypic screening strategy using isogenic KO cell lines that simultaneously evaluates permeability, target dependence, and anticancer activity under physiologically relevant conditions. This approach led us to identify HBC-8, whose anticancer effects depend on the presence of MAP3K1 and CRBN and operate through mechanisms that extend beyond loss of MAP3K1 protein. Our comparative proteomic analysis reveals that HBC-8 treatment shares only 89 altered proteins with *MAP3K1* KO (11.3%) and 11 proteins with *CRBN* KO (1.4%). This indicates that over 85% of HBC-8’s proteomic signature represents unique cellular perturbations not achievable through genetic knockout alone. These findings question the prevailing assumption that the degrader’s therapeutic effects primarily result from target protein elimination and instead suggest that these HBCs induce distinct network-level alterations. Importantly, these broad effects reflect the combined consequences of MAP3K1 degradation and the activity of the CRBN-recruiting moiety, such as neo-substrate degradation and potential CUL4 rewiring. Selectivity for malignant cells is nonetheless maintained: HBC-8 activity strictly requires both CRBN and MAP3K1, spares normal hematopoietic (WBC) colony formation at active concentrations (Fig. S3a), and produced no observable toxicity in tumor-bearing mice (Fig. 8g). HBC-8 operates through molecular mechanisms that extend beyond MAP3K1 degradation, which is further supported by the ~100-fold difference in the IC_50_ and DC_50_ values. Mechanistically, HBC-8 showed low-nM potency in 267 of 869 cancer cell lines (31%) across more than 20 cancer types, with sensitivity correlating with CRBN and MAP3K1 expression levels. Beyond target-dependent cytostatic and cytotoxic effects, HBC-8 disrupts MAP3K1-mediated signaling networks that are dysregulated in cancer progression. Treatment with HBC-8 reduced phosphorylation of downstream effectors ERK and IKKβ while increasing JNK phosphorylation, consistent with MAP3K1’s role as an upstream regulator of MAPK and NF-κB pathways [25–29]. Sustained JNK activation likely contributes to HBC-8-induced apoptosis, as prolonged JNK signaling promotes mitochondrial-mediated cell death. The observed cytostatic effects could be attributed to reduced ERK phosphorylation. The modest changes in total IKKβ and ERK levels are an indirect consequence of HBC-8 attenuating global protein synthesis: when translation is suppressed, short-lived proteins are depleted first, so high-turnover kinases such as IKKβ and ERK show reduced total levels, whereas some proteins such as AKT are unchanged. This preferential loss of short-lived proteins under translational suppression is well established [76].

We also show that MAP3K1 forms a baseline complex with CRBN, CUL4A, and DDB1. This finding suggests that HBC-8 engages pre-existing protein–protein interaction networks rather than creating new associations. Our genome-wide CRISPR screen using HBC-8 in NALM6 cells supports this mechanism, identifying several genes involved in the CUL4 E3 ligase system, such as CRBN, DDB1, NEDD8, and CUL4A, as essential for HBC-8’s anticancer activity. The importance of this E3 ligase complex for HBC-8 activity is further highlighted by several CUL4 regulatory proteins, such as NEDD8, COP9, and ubiquitin-related proteins, that had high gene dependency scores. As noted above, the CRBN-CUL4 system is a dynamic regulatory hub whose perturbation can trigger cascading, therapeutically relevant effects [22]. Furthermore, multi-omics correlation analysis using AUC of HBC-8 identified CRBN-associated expression, protein levels, and copy number as the strongest predictors of sensitivity to HBC-8. These observations are likely attributable to the pomalidomide fragment, the E3 ligase ligand, which recruits CRBN [6, 65, 67], in HBC-8, indicating the importance of E3 ligase recruitment for HBC-8 efficacy. Conversely, ABC transporter family proteins emerged as key resistance drivers, consistent with their known role in drug efflux and PROTAC resistance [77].

The genome-wide CRISPR screen identified protein translation machinery as another critical vulnerability associated with HBC-8 treatment, with multiple translation initiation factors (EIF4A1, EIF4B, EIF3E, EIF4G1) emerging as dependencies. Importantly, these translation factors showed minimal protein-level changes in our proteomic analysis, suggesting they represent downstream vulnerabilities rather than direct degradation targets. In parallel, HBC-8 directly degrades the CRBN neo-substrate GSPT1/2, a translation termination factor, in our HCT116 proteomics (Fig. 6i–j). Although GSPT1 was not itself a screen hit, its degradation represents an independent perturbation of the translation apparatus, converging with the screen dependencies on protein synthesis as a point of vulnerability. Notably, pro- and anti-apoptotic regulators did not emerge as resistance determinants in the screen, consistent with HBC-8–induced cell death being a downstream output, engaged in part through the sustained JNK activation described above, rather than a discrete genetic dependency. The convergent evidence from multiple orthogonal approaches suggests that the ternary complex MAP3K1::HBC-8::CRBN disrupts protein synthesis networks through indirect mechanisms, potentially involving altered stoichiometry of regulatory complexes or subcellular relocalization of key factors. Our identification of translation machinery as a critical vulnerability exemplifies that the therapeutic effects associated with HBCs involve system-level disruptions in addition to primary target degradation. This finding aligns with growing evidence that HBC efficacy often depends on perturbation of interconnected cellular processes rather than simple protein elimination. For example, the anticancer effects associated with ARV-771 result from BET protein degradation and downstream transcriptional network disruption that affects AR and c-MYC signaling [78]. Similarly, dBET1 exerts its therapeutic effects through coordinated suppression of inflammatory networks involving multiple cytokines and stress-response mediators [79]. These examples, along with translation machinery dependencies presented here, suggest that the observed therapeutic effects associated with HBCs arise from disrupting cellular networks through ternary complex formation, creating context-dependent vulnerabilities that extend beyond primary target elimination.

We propose that HBC-8 likely stabilizes specific conformational states or alters exchange kinetics to favor stoichiometric rebalancing of the MAP3K1-CRBN-CUL4 complexes (Fig. S9). Sustained HBC-8 presence perturbs the dynamic CUL4-CRBN E3 ligase equilibrium, which typically undergoes rapid substrate-receptor exchange. Given the involvement of diverse CUL4 complexes in critical cellular processes ranging from DNA repair to cell cycle control [56, 64, 80, 81], even subtle perturbations in complex assembly, subcellular localization, or substrate-receptor exchange rate could trigger cascading effects that disrupt cellular homeostasis, leading to the observed proteome-wide changes that the genetic KOs cannot recapitulate. This proposed mechanistic complexity highlights that HBC’s therapeutic effects depend on the unaccounted multifunctional properties, in addition to target elimination, to drive anticancer efficacy.

The demonstration that HBC-8 operates through network perturbation mechanisms distinct from genetic knockouts argues for a fundamental shift from degradation-focused to phenotype-based screening strategies. Viability-based screening in isogenic knockouts provides a framework for identifying therapeutically relevant compounds that act through target- and E3-ligase-dependent mechanisms while capturing the full complexity of cellular network responses that conventional approaches miss. Through chemical genetic screening, genome-wide dependency profiling, proteome-wide analysis, and large-scale correlation studies, we report that HBC-8 achieves therapeutic efficacy by disrupting interconnected cellular networks involving cell cycle control and protein synthesis. The in vivo efficacy observed in the HBC-8-treated ovarian cancer model further supports the argument that phenotypic screening using isogenic cell lines to identify hits will minimize false negatives that rely on target degradation but are target-dependent. The study reported here identified a first-in-class HBC that exhibits MAP3K1- and CRBN-dependent anticancer effects.

Several limitations should be noted. First, our proteomic comparison contrasts knockout cells that underwent chronic deletion and culture selection, which can acquire compensatory adaptations, with acutely treated cells that additionally mount transient stress responses; the two conditions are not fully equivalent, and part of the > 85% unique HBC-8 signature may reflect this difference rather than degrader-specific network effects alone. Second, HBC-8’s activity is not restricted to MAP3K1: its CRBN handle degrades neo-substrates (e.g., GSPT1) and engages the CUL4 machinery, so its phenotype reflects a combination of MAP3K1 loss and broader CRBN-dependent effects. Third, several sensitivity relationships derive from correlative cell-line profiling; although corroborated by isogenic and genome-wide genetic data, they remain associative. Finally, we established in vivo efficacy in a single orthotopic ovarian model, and the phenotypic screening approach, along with its reliance on the CRBN-CUL4 system, will require validation across additional target proteins and E3-ligase systems. Our ongoing work addresses several of these points, incorporating temporal proteomics to distinguish direct degradation from secondary responses and testing whether the translation-machinery dependencies are unique to HBC-8 or a common feature of network-hub–targeting HBCs. Although our genetic and pharmacological data indicate that HBC-8 promotes MAP3K1 degradation through a CRL4-dependent, proteasomal mechanism, a full biochemical and biophysical characterization of HBC-8–induced MAP3K1 ubiquitination remains an important direction for future work. These findings have important implications for future HBC design and screening strategies, emphasizing the need to consider network effects alongside traditional degradation metrics.

Collectively, this work provides proof of concept that therapeutically relevant HBCs achieve anticancer effects by perturbing complex signaling networks that extend beyond primary target degradation. It also shows that viability-based screening in isogenic cell lines can identify compounds whose activity is target- and E3-dependent. The framework presented here offers a practical basis for developing next-generation HBCs selected for target dependence in addition to degradation.

## Materials and methods

### Cell lines and culture conditions

HCT116 wild-type, HCT116* MAP3K1* KO (generated at MSKCC genome editing core), HCT116 *CRBN* KO cells (generated at UNMC genome editing core), and SKOV3-luciferase cells were cultured in McCoy’s 5 A medium with 10% FBS and 1% Penicillin–Streptomycin. T47D cells (from Dr. Vimla Band’s Lab, UNMC) were cultured in RPMI medium with 10% FBS, 1% Penicillin–Streptomycin, and 100 units/mL insulin. NCIH1048 cells were cultured in DMEM/F12 medium with 5% FBS, 0.005 mg/mL insulin, 0.01 mg/mL transferrin, 30 nM sodium selenite, 10 nM hydrocortisone, 10 nM beta-estradiol, 2 mM L-glutamine (for final concentration of 4.5 mM), and 1% Penicillin–Streptomycin. Cells were maintained in a humidified 5% CO_2_ incubator at 37 °C.

For cell line validation, HCT116 wild-type, HCT116 *CRBN* KO, T47D, and MCF7 cells (1 × 10^6^) were collected during a routine passage, washed with PBS, pelleted by centrifugation (2.5 × 10^3^ rpm, 5 min), and submitted to the UNMC Human DNA Identification Laboratory for STR profiling; cell lines with ≥ 80% match to the ATCC database (www.atcc.org) were considered authentic. HCT116 wild-type and NCIH1048 were obtained directly from ATCC, which authenticates its cell lines by STR profiling, and were used at low passage. The HCT116 MAP3K1 KO line was generated from ATCC-authenticated parental HCT116 and validated by target-site (plasmid) sequencing reported previously [46].

### Viability screening assay

HCT116 WT, HCT116 *CRBN* KO, and HCT116 *MAP3K1* KO cells (4,000 cells in 90 µL media) were plated in 96-well plates and allowed to adhere overnight. HBC library working solutions were prepared from 10 mM DMSO stocks, and 10 µL was added to each well to yield a final concentration of 1 μM in 1% DMSO. At treatment start, 10 µL PrestoBlue was added to control wells and incubated for 15 min at 37 °C. Fluorescence (Ex 560, Em 590) was measured on a plate reader (SpectraMax M5e) to determine T0. After 72 h, 10 µL PrestoBlue was added to the remaining wells, incubated for 15 min, and fluorescence was measured to determine T100. Cell viability was calculated as [(Fluorescence – T0)/(T100 – T0)]. Data were plotted using GraphPad Prism 10.5.0, and IC_50_ values were determined using four-parameter logistic curve-fitting.

### Cell growth inhibition assays

Cells (T47D and NCIH1048: 6,000 cells/well; HCT116 and SKOV3: 4,000 cells/well) were plated in 90 µL media in 96-well plates and allowed to adhere overnight. Compound working solutions were prepared from 10 mM DMSO stocks in 6- 8 dilutions and added in triplicate (10 µL, final 1% DMSO). At treatment start, 10 µL PrestoBlue was added to control wells, incubated for 15 min at 37 °C, and fluorescence (Ex 560, Em 590) was measured to determine T0. Plates were incubated for 72–120 h, then 10 µL PrestoBlue was added to the remaining wells, incubated for 15 min, and fluorescence was measured to determine T100. % Growth was calculated as [(Fluorescence – T0)/(T100 – T0)] × 100. Data were plotted using GraphPad Prism 10.5.0, and IC_50_ values were determined using four-parameter logistic curve-fitting.

### Western blot analyses

Cells were washed with cold 1X PBS and lysed using RIPA buffer supplemented with sodium orthovanadate, sodium fluoride, β-glycerophosphate, and 1 mM PMSF. Protein was quantified using BCA Protein Assay. 40 μg protein was separated by SDS-PAGE on 7.5% or 4%–15% gradient gels at 120 V for ~80 min in 1X TRIS–Glycine SDS buffer, then transferred to PVDF membranes using semi-dry transfer at 18 V for 35 min. Membranes were blocked with 5% non-fat dry milk (or 5% BSA for phospho-antibodies) in 1X TBST for 1 h at room temperature, then incubated with primary antibodies at a 1:1000 dilution (unless otherwise noted) in 5% milk or 5% BSA in 1X TBST overnight at 4 °C. Primary antibodies used: anti-MAP3K1, anti-pERK1/2, anti-ERK1/2, anti-pIKKα/β, anti-IKKα/β, anti-pJNK, anti-JNK, anti-DUSP4, anti-pAKT (T308), anti-pAKT (S473), anti-AKT, anti-pMEK1/2, anti-MEK, anti-CRBN, anti-Vinculin, anti-α-tubulin, anti-HSP90, anti-CDK4, anti-CDK2, anti-CDK12, anti-CDK1, anti-CDK9, anti-MAP2K6, anti-P38, anti-ASK1, anti-FAK, anti-DLK1, anti-TRAF1, and anti-AMPK. Proteins were visualized using a molecular weight ladder. After washing with 1X TBST (3 × 10 min), membranes were incubated with HRP-conjugated secondary antibodies (anti-rabbit or anti-mouse) at 1:2000 dilution in 5% milk or 5% BSA in 1X TBST for 1 h at room temperature, followed by washing (3 × 10 min). Protein expression was detected using ECL Prime and developed using ChemiDoc. We performed Western blots in multiple biological replicates.

### Pharmacokinetics studies

Pharmacokinetic studies (30 mg/kg oral and intravenous dosing) were performed following previously reported methods^9^. Sample analysis was performed by LC–MS/MS (SCIEX QTRAP 4000 System). Pharmacokinetic parameters were determined using non-compartmental analysis in WinNonlin 8.3.

### Xenograft studies

SKOV3-luciferase cells (5 × 10^6^) were suspended in PBS:Matrigel (1:1, v/v) and injected intraperitoneally into 5- 6-week-old female athymic nude mice. When total bioluminescent flux reached ≥ 1 × 10^8^ photons/sec, mice were randomized and treated for 4 weeks with vehicle or HBC-8 at 7.5 or 15 mg/kg (IP, six days/week). Vehicle composition: 5% DMSO, 40% PEG-400, 10% Cremophor, 45% Tris buffer (10 mM, pH 8.0, 150 mM NaCl). Before imaging, animals received D-luciferin via IPT at 10 μL/g from a 15 mg/mL stock (~150 mg/kg). Tumor burden was measured weekly using the IVIS Spectrum system, and quantitative analyses were performed with Living Image v4.8.2. Ex vivo bioluminescence imaging was performed at the endpoint on excised peritoneal organs. Animals were housed in pathogen-free facilities. All procedures were approved by UNMC IACUC. Full animal husbandry, randomization, dosing, humane-endpoint, euthanasia, and immunohistochemistry details, reported in accordance with the ARRIVE 2.0 guidelines, are provided in Supplementary Methods.

### Statistical analysis

Data are presented as mean ± SEM from at least three independent biological replicates. Statistical analyses were performed using GraphPad Prism 10.5.0. IC_50_ values were calculated using four-parameter logistic curve-fitting. Pairwise comparisons used unpaired two-tailed t-tests; multiple comparisons used one-way ANOVA with Tukey’s post-hoc test. The Mann–Whitney test was used for unequal sample sizes. Proteomics data were analyzed using Welch’s t-test. We used Fisher’s exact test for gene dependency analysis. We analyzed CRISPR screen data using the modified CRANKS algorithm. Statistical significance: *p < 0.05, **p < 0.01, ***p < 0.001. Specific tests and sample sizes are in the figure legends.

## Supplementary Information

Supplementary Material 1.

## Data Availability

The experimental data sets generated and/or analyzed during the current study are available from the corresponding author upon request. The mass spectrometry proteomics data have been deposited in the ProteomeXchange Consortium via the PRIDE partner repository with the dataset identifier PXD070052. Genome-wide CRISPR screen data are available at GEO accession number: GSE347763. Requests for materials should be addressed to the corresponding author Amarnath Natarajan (nataraah@ucmail.uc.edu).
