# Supplementary material for: Chemical genetic screens combine target dependence with efficacy: discovery of a MAP3K1 degrader (HBC-8) with anticancer activities
Source: Mol Biomed. 2026 Oct 28;7:230. doi: 10.1186/s43556-026-00632-z (PMC13719937; doi:10.1186/s43556-026-00632-z)
Supplement: Supplementary file 1 [file 43556_2026_632_MOESM1_ESM.docx]

**Title:**	Chemical genetic screens combine target dependence with efficacy: Discovery of a MAP3K1 degrader (HBC-8) with anticancer activities

**Authors**: ^1,2^Lelisse T. Umeta, ^2^Jayapal Reddy Mallareddy, ^2^Sarbjit Singh, ^1,2^Karan Vishwakarma, ^1,2^Smitha Kizhake, ^1,2^Wangbin Wu, ^2^Amritha G. Kizhake, ^2^Lidia Boghean, ^1,2,3^Suchita Vishwakarma, ^1,2,3^Mihir Kachhia, ^2^AJ Sallee, ^4^Vaishnavi Sunil Joshi, ^2^Katelyn L. O'Neill, ^2^Lisa Humphrey-Brattain, ^4^Linda Chee, ^5^Matthew G. Rees, ^5^Melissa M. Ronan, ^5^Jennifer A. Roth, ^6^Andrew Chatr-aryamontri, ^6^Thierry Bertomeu, ^2^Kabhilan Mohan, ^2^Yuanhong Chen, ^4^Kyle Hewitt, ^2^Grinu Mathew, ^2^Adrian Black, ^4^Gargi Ghosal, ^2^Xu Luo, ^2^Jixin Dong, ^2^Nicholas T. Woods, ^1,2,3,4^Amarnath Natarajan*

**Affiliation**:

^1^Division of Pharmaceutical Sciences, James L. Winkle College of Pharmacy, University of Cincinnati, Cincinnati, OH 45267 USA;

^2^Eppley Institute for Cancer Research, University of Nebraska Medical Center, Omaha, NE 68198 USA;

^3^Department of Pharmaceutical Sciences, University of Nebraska Medical Center, Omaha, NE 68198 USA;

^4^Department of Genetics, Cell Biology and Anatomy, University of Nebraska Medical Center, Omaha, NE 68198 USA;

^5^Broad Institute of MIT and Harvard, Cambridge, MA 02142 USA;

^6^The ChemoGenix CRISPR screening platform of the Institute for Research in Immunology and Cancer (IRIC), Université de Montréal, Montréal, QC, H3T 1J4, Canada.

*Corresponding author

Amarnath Natarajan

[nataraah@ucmail.uc.edu](mailto:nataraah@ucmail.uc.edu)

**Supplementary Figure 1: Screening of MAP3K1-targeted HBC library in T47D and MCF7, luminal B breast cancer cell lines:** (a) Viability screening of the quinoxaline-based HBC library in T47D cells treated at 100 nM for 120 hours. Data are presented as mean ± SEM, n = 3 biological replicates. (b) Viability screening of the quinoxaline-based HBC library in MCF7 cells treated at 10 μM for 72 hours. Data are presented as mean ± SEM, n = 3 biological replicates. Treatment conditions were optimized based on cell line doubling time and sensitivity, which correlate with MAP3K1 and CRBN expression levels (see Fig. 4e–g).

**Supplementary Figure 2: Genetic and Chemical-based mechanism of action validation of HBC-8:** (a) Cell cycle distribution in HeLa cells treated with DMSO or HBC-8 (1 μM) for 24 hours. Numbers indicate the percentage of cells in each phase. Data are individual data points, n = 3 biological replicates (**p < 0.001). (b) Dose-response curves of HBC-8, HBC-8C and HBC-23 in CRC organoids (Q20-70T) treated with HBC compounds for 120 hours. Data are presented as individual data points, n = 4 biological replicates. (c) Cell cycle distribution in HCT116 cells treated with DMSO or 1 μM of HBC-8 and HBC-8C for 24 hours. Numbers indicate the percentage of cells in each phase. Data are presented as mean ± SEM, n = 3 biological replicates (***p < 0.001; ns, not significant). (d) Caspase 3/7 activity in HCT116 cells treated with DMSO, HBC-8 (1 μM), HBC-8C (5 μM), or HBC-23 (5 μM) for 24 hours. Data are presented as fold change (FC) relative to DMSO control, mean ± SEM, n = 3 biological replicates (*p < 0.05; ns, not significant).

**Supplementary Figure 3: Large-Scale Cell Line Profiling of HBC-8:** (a) Colony formation assay of CD34+ hematopoietic progenitor cells treated with HBC-8 for 14 days. Numbers in bars indicate the mean colony count. Data are presented as mean ± SD, n=5 for DMSO and 0.1 µM, n=2 for 0.025 µM and 0.05 µM biological replicates. Statistical analysis by unpaired t-test (*p < 0.05 compared to DMSO control). (b) A bar graph showing the distribution of 869 cancer cell lines across 28 cancer types included in the HBC-8 PRISM viability screen. (c) Correlation between *CRBN* (black) or *MAP3K1* (magenta) mRNA expression and HBC-8 sensitivity across cancer cell lines profiled in the PRISM viability screen. Each point represents an individual cell line, with DepMap expression values plotted as Log₂(TPM + 1) against Log₂(HBC-8 IC_50_); solid lines show linear regression fits. Correlation coefficients (R) and p-values are tabulated below the plot. *CRBN* expression was significantly negatively correlated with HBC-8 IC_50_ (higher expression, greater sensitivity; R = −0.24, p < 0.0001), whereas *MAP3K1* expression showed a weaker, non-significant association (R = −0.07, p = 0.0562). (d) Top 10 significant correlation analysis using Depmap dataset for shRNA, metabolomics, and repurposed compound biomarkers (q values < 0.05) of sensitivity to HBC-8 (log_2_(AUC)).

**Supplementary Figure 4: Mechanism of HBC-8-induced MAP3K1 degradation:** (a) Growth inhibition assay in NCIH1048 cells treated with HBC-8 for 72 hours. Data are presented as mean ± SEM (n = 3). (b) Western blot analysis showing MAP3K1 protein levels in NCIH1048 cells treated with HBC-8 for 12 hours (n=4). (c) DC_50_ curve using ImageJ quantified MAP3K1 protein levels normalized to DMSO from (b) (n=4) biological replicates. (d) Western blot analysis (n=1) of NCIH1048 cells co-treated with HBC-8 (10 nM) and/or iMAP3K1 (100 nM) or Pomalidomide (100 nM) for 12 hours. (e) Western blot analysis (n=1) of NCIH1048 cells co-treated with HBC-8 (10 nM) and/or MG-132 (100 nM) for 12 hours. (f) Western blot analysis (n=1) of NCIH1048 cells co-treated with HBC-8 (10 nM) and/or MLN-4924 neddylation inhibitor (100 nM) for 12 hours. (g) Growth inhibition assay in HeLa cells treated with HBC-8, MLN-4924, or HBC-8 + MLN-4924 (1:1 ratio). Data are presented as mean ± SEM, n = 3 biological replicates. (h) Western blot analysis showing PARP and cleavage of PARP in NCIH1048 cells treated with HBC-8 for 12 hours. (i) Time-course analysis of apoptosis induction in NCIH1048 cells treated with HBC-8 (10 nM). Data presented as fold change ± SEM, n = 3, in apoptosis induction relative to 0 hour at indicated time points. (j) Western blot analysis of NCIH1048 cells co-treated with HBC-8 (10 nM) and/or Z-VAD (10 μM) for 12 hours. HSP90 is used as a loading control for each immunoblot panel. Molecular weight markers (kDa) are indicated on the left.

**Supplementary Figure 5: Effect of HBC-8 on MAP3K1 downstream signaling:** (a) MAP3K1-mediated downstream signaling pathways. *Created in BioRender. Vishwakarma, K. (2026)* [*https://BioRender.com/01wvi5h*](https://BioRender.com/01wvi5h). (b) Western blot analysis of proteins in MAP3K1-mediated signaling in T47D cells treated with HBC-8 (100 nM) (n=3), (c) Western blot analysis of Total protein levels of various kinases in T47D cells treated with HBC-8 (100 nM) (n=1), (d) Western blot analysis of CUL4-associated proteins in T47D cells treated with HBC-8 (100 nM) (n=1), (e) Immunoprecipitation of MAP3K1 protein complexes in HCT116 cells treated with HBC-8 (1 μM) or left untreated (UT). IP using MAP3K1 antibody or IgG control and immunoblot for the indicated proteins (n = 2). α-tubulin, and β-actin are loading controls for each immunoblot panel. Molecular weight markers (kDa) are indicated on the left.

**Supplementary Figure 6: Proteome-wide profiling of HBC-8:** Gene Ontology (GO) enrichment analysis of differentially expressed proteins from a proteomics study using Enrichr. Bar plots show significantly enriched in GO Biological Processes 2026 for downregulated and upregulated proteins in (a) HCT116 WT cells treated with HBC-8 (0.5 μM), (b) HCT116 WT cells vs *MAP3K1* KO cells baseline, and (c) HCT116 WT cells vs *CRBN* KO cells baseline. Venn diagrams show protein overlaps between HBC-8 treatment in (d) HCT116 WT vs *MAP3K1* KO (e) HCT116 WT vs *CRBN* KO. Bar plots show significantly enriched biological processes for proteins altered by (f) HBC-8 treatment in *MAP3K1* KO cells, (g) HBC-8 treatment in *CRBN* KO cells. GO analysis with FDR < 0.05. Volcano plots showing differentially expressed proteins (fold change ≥ 2, *p* < 0.05) in HCT116 cells: (h) MAP3K1 E3 ligase target genes in WT cells treated with HBC-8 versus DMSO. (i) The same target gene set in *MAP3K1* KO versus WT cells. n = 3 biological replicates.

**Supplementary Figure 7: Genome-wide profiling of HBC-8:**(a) Viability assay in NALM6 cells treated with HBC-8 for 72 hours with IC_50_ = 0.35 μM. Data are presented as individual data points from quadruplicate measurements across (n=2) independent experiments. (b) Volcano plot showing differentially expressed proteins (fold change ≥2, p<0.05) in HCT116 WT cells treated with HBC-8 versus DMSO, highlighting the top CRANKS-score genes; these dependency genes showed no significant change at the protein level. Mass spectrometry was performed on n=3 biological replicates; statistical analysis by unpaired t-test with unequal variance. (c) Extended STRING network analysis showing MAP3K1's interactions with rescue genes from both the translation machinery (green box) and ubiquitin-proteasome (pink box) clusters. Key interactions of MAP3K1 with FKBP5, DDB1, and COPS6 were identified. Nodes represent proteins, with colored nodes indicating query proteins (our identified genes) and white nodes showing second shell interactors. Filled nodes have known or predicted 3D structures. Edges represent different types of evidence known interactions from curated databases (light blue) and experimental data (magenta), predicted interactions from gene neighborhood (green), gene fusions (red), and gene co-occurrence (dark blue), plus text mining (yellow), co-expression (black), and protein homology (light blue).

**Supplementary Figure 8: Evaluation of HBC-8 efficacy in SKOV3-luc intraperitoneal tumor model using bioluminescent imaging:** (a) Representative Dorsal bioluminescent images of SKOV3-luc tumor-bearing mice treated with vehicle or HBC-8 (7.5 mg/kg or 15 mg/kg) at the indicated time points. Ex vivo (dorsal) IVIS imaging of mice treated with vehicle or HBC-8 (7.5 mg/kg or 15 mg/kg). Data represent mean ± SEM (vehicle, n = 5; HBC-8 7.5 mg/kg, n = 4; HBC-8 15 mg/kg, n = 5). (b) Quantification of average radiance (p/s/cm2/sr) over 28 days. Data are presented as individual animal traces (vehicle, n = 5; HBC-8 7.5 mg/kg, n = 4; HBC-8 15 mg/kg, n = 5). (c) Macroscopic examination of peritoneal tumor burden at necropsy. (d) Histological and immunohistochemical analysis of tumor tissues. H&E staining (top), Ki-67 (middle), and cleaved caspase-3 (bottom) in tumors from vehicle- and HBC-8–treated mice. Scale bar, 100 µm. D-HBC-8 / V-HBC-8 (dorsal/ventral) and D-C / V-C (vehicle control)

**Supplementary Figure 9**: Schematics showing possible mechanism of action of HBC-8. *Created in BioRender. Umeta, L. (2026)* [*https://BioRender.com/3x6ol9c*](https://BioRender.com/3x6ol9c)*.*

**Supplementary Table 1: Description of quinoxaline-based HBCs linker composition, linker length, and respective substitutions.**

| **HBC** | **R** | **X** | **Y** | **Z** | **Linker** | **M. Wt.** | **tPSA** | **cLogP** |
| --- | --- | --- | --- | --- | --- | --- | --- | --- |
| **HBC-1** | CF3 | NH | C=O | H  |  | 930.90 | 243.05 | 2.18 |
| **HBC-2** | CF3 | NH | C=O | H |  | 754.69 | 206.13 | 2.64 |
| **HBC-3** | CF3 | NH | C=O | H |  | 842.79 | 224.59 | 2.53 |
| **HBC-4** | CF3 | NH | C=O | H |  | 796.77 | 206.13 | 3.48 |
| **HBC-5** | CF3 | NH | C=O | H |  | 838.85 | 206.13 | 5.07 |
| **HBC-6** | CF3 | NH | C=O | H |  | 824.82 | 206.13 | 4.54 |
| **HBC-7** | CF3 | NH | C=O | H |  | 886.85 | 233.82 | 2.35 |
| **HBC-8** | CF3 | NH | C=O | H |  | 768.71 | 206.13 | 3.0 |
| **HBC-8C** | CF3 | NH | C=O | CH3 |  | 782.74 | 197.34 | 3.66 |
| **HBC-9** | CF3 | NH | CH2 | H |  | 828.81 | 207.52 | 2.31 |
| **HBC-10** | CF3 | NH | CH2 | H |  | 784.76 | 198.29 | 2.48 |
| **HBC-11** | CF3 | NH | C=O | H |  | 798.74 | 215.36 | 2.70 |
| **HBC-12** | CF3 | O | C=O | H |  | 944.88 | 260.12 | 1.72 |
| **HBC-13** | CF3 | O | C=O | H |  | 840.78 | 232.43 | 1.89 |
| **HBC-14** | CF3 | O | C=O | H |  | 812.72 | 232.43 | 2.01 |
| **HBC-15** | CF3 | O | C=O | H |  | 755.67 | 203.33 | 2.70 |
| **HBC-16** | Br | NH | C=O | H |  | 853.69 | 224.59 | 2.73 |
| **HBC-17** | Br | NH | C=O | H |  | 941.81 | 243.05 | 2.38 |
| **HBC-18** | Br | O | C=O | H |  | 955.78 | 260.12 | 1.92 |
| **HBC-19** | Br | O | C=O | H |  | 851.68 | 232.43 | 2.09 |
| **HBC-20** | Br | NH | C=O | H |  | 809.64 | 215.36 | 2.90 |
| **HBC-21** | Br | NH | C=O | H |  | 897.74 | 233.82 | 2.55 |
| **HBC-22** | Br | NH | C=O | H |  | 765.58 | 206.13 | 2.84 |
| **HBC-23** | Br | NH | C=O | H |  | 779.61 | 206.13 | 3.20 |

**Supplementary Table 2: Phenotypic screening results with statistical analysis (mean, SD, and p-value)**

| **Compound** | **WT mean** | **WT SD** | ***CRBN* KO mean** | ***CRBN* KO SD** | **p (WT vs *CRBN* KO)** | ***MAP3K1* KO mean** | ***MAP3K1* KO SD** | **p (WT vs *MAP3K1* KO)** |
| --- | --- | --- | --- | --- | --- | --- | --- | --- |
| HBC-1 | 1.239 | 0.116 | 1.187 | 0.228 | 0.626 | 0.921 | 0.047 | 3.0E-03 |
| HBC-2 | 1.177 | 0.12 | 1.086 | 0.233 | 0.413 | 0.988 | 0.034 | 0.036 |
| HBC-3 | 1.228 | 0.163 | 1.201 | 0.183 | 0.796 | 1.019 | 0.035 | 0.072 |
| HBC-4 | 1.242 | 0.075 | 1.062 | 0.075 | 0.043 | 0.865 | 0.003 | 6.7E-03 |
| HBC-5 | 0.927 | 0.128 | 1.138 | 0.241 | 0.087 | 1.017 | 0.034 | 0.284 |
| HBC-6 | 0.725 | 0.122 | 1.097 | 0.243 | 7.2E-03 | 1.015 | 0.037 | 5.9E-03 |
| HBC-7 | 1 | 0.205 | 0.963 | 0.249 | 0.784 | 0.957 | 0.127 | 0.754 |
| **HBC-8** | **0.229** | **0.108** | **0.777** | **0.15** | **2.8E-05** | **1.014** | **0.039** | **7.0E-06** |
| HBC-9 | 1.126 | 0.048 | 1.075 | 0.194 | 0.550 | 1.059 | 0.025 | 0.061 |
| HBC-10 | 1.126 | 0.163 | 1.043 | 0.223 | 0.478 | 1.043 | 0.042 | 0.432 |
| HBC-11 | 0.802 | 0.19 | 1.067 | 0.232 | 0.056 | 1.045 | 0.031 | 0.070 |
| HBC-12 | 1.189 | 0.153 | 1.187 | 0.182 | 0.988 | 1.048 | 0.016 | 0.170 |
| HBC-13 | 1.079 | 0.183 | 1.124 | 0.162 | 0.660 | 1.039 | 0.026 | 0.731 |
| HBC-14 | 1.192 | 0.355 | 1.165 | 0.224 | 0.877 | 1.097 | 0.033 | 0.669 |
| HBC-15 | 1.229 | 0.188 | 1.107 | 0.24 | 0.350 | 0.996 | 0.002 | 0.076 |
| HBC-16 | 1.042 | 0.118 | 1.052 | 0.168 | 0.906 | 1.043 | 0.024 | 0.989 |
| HBC-17 | 1.093 | 0.091 | 1.009 | 0.153 | 0.278 | 1.026 | 0.016 | 0.261 |
| HBC-18 | 0.968 | 0.329 | 0.877 | 0.172 | 0.563 | 1.005 | 0.026 | 0.855 |
| HBC-19 | 1.196 | 0.191 | 1.03 | 0.207 | 0.179 | 1.028 | 0.017 | 0.185 |
| HBC-20 | 1.081 | 0.187 | 1.053 | 0.214 | 0.812 | 1.005 | 0.031 | 0.520 |
| HBC-21 | 1.275 | 0.104 | 1.141 | 0.179 | 0.144 | 1.048 | 0.033 | 9.0E-03 |
| HBC-22 | 1.371 | 0.422 | 1.077 | 0.184 | 0.149 | 1.057 | 0.022 | 0.254 |
| HBC-23 | 0.927 | 0.16 | 1.127 | 0.16 | 0.069 | 0.969 | 0.144 | 0.755 |

**Supplementary Table 3: Summary of differentially expressed proteins across mass spectrometry experimental conditions.**

|  | Up regulated | Downregulated |
| --- | --- | --- |
| WT + HBC 8 | 447 | 340 |
| *MAP3K1* KO | 217 | 45 |
| *CRBN* KO | 9 | 46 |
| *MAP3K1* KO + HBC 8 | 73 | 534 |
| *CRBN* KO + HBC 8 | 17 | 84 |

**SUPPLEMENTARY METHODS**

**Heterobifunctional Compounds (HBC) Synthesis**

All reagents were purchased from commercial sources and were used without further purification. Flash chromatography was carried out on silica gel (200–400 mesh). Thin-layer chromatography (TLC) was run on a pre-coated ANALTECH uniplate and observed under UV light at 254 nm and with basic potassium permanganate dip. Column chromatography was performed with silica gel (230-400 mesh, grade 60). ¹H NMR (400 MHz) and ¹³C NMR (100 MHz) spectra were recorded in chloroform-d or DMSO-d6 on a Bruker-400 spectrometer (DMSO-d6 was 2.50 ppm for ¹H and 39.55 ppm for ¹³C, and chloroform-d (CDCl3) was 7.26 ppm for ¹H and 77.23 ppm for ¹³C. Proton and carbon chemical shifts were reported in ppm relative to the residual solvent proton and carbon signal. The data are presented as follows: chemical shift, multiplicity (s = singlet, d = doublet, t = triplet, q = quartet, p = pentet, m = multiplet and/or multiple resonances), coupling constant in hertz (Hz), and integration. The purity of final compounds was determined by analytical HPLC and was found to be ≥95% pure. HPLC was performed on a Waters Alliance 2690 system equipped with a Waters 2996 photodiode array detector and an auto-sampler under the following conditions: column, Phenomenex Luna-2 RP-C18 (5 µm, 4.6 mm × 250 mm, 120 Å); solvent A, H_2_O containing 0.1% formic acid (FA); solvent B, CH3CN containing 0.1% FA; gradient, 60% B to 100% B over 8 minutes followed by 100% B over 6 min; injection volume, 25 µL; flow rate, 1 mL/min. Retention times and purity data for each target compound are provided in the Experimental Section. The purified compound was further confirmed by MS analysis using a Quattro Micro triple quadrupole mass spectrometer using the electron spray ionization (ESI) technique and as a TOF mass analyzer. Compound 41 was synthesized by using the reported procedure. Detailed synthetic procedures and NMR characterization data are provided in Chemistry Supplementary Information.

**Immunoprecipitation (IP)**

HCT116 WT cells were plated at 80% confluency and treated with 1 µM HBC-8 for 2 hours. Cells were collected in PBS and centrifuged at 1,000 rpm for 5 minutes at 4°C. The pellet was lysed in 1 mL NETN lysis buffer, transferred to a 1.5 mL Eppendorf tube, and incubated for 20 minutes on a rocker at 4°C. Lysate was centrifuged at 13,000 rpm for 15 minutes at 4°C. The supernatant was divided into 3 aliquots: 1% Input (+25 µL SDS dye; stored at -20°C), IgG control, and IP sample. 1 µL IgG (1:600 dilution) was added to 300 µL of lysate for control, and 1 µL anti-MAP3K1 was added to 300 µL lysate for IP. IgG and IP lysates were incubated with antibodies on a rocker overnight (~15 hours) at 13 rpm at 4°C. After 15 hours, 20 µL protein A/G beads were added and incubated on a rocker at 4°C for 2 hours. Beads were washed 5 times with 1 mL NETN by centrifuging at 1,000 rpm for 5 minutes at 4°C and eluted in 50 μL SDS dye. Samples (Input, IgG, and IP) were denatured for 5 minutes at 95°C, followed by Western Blot analysis.

**Caspase 3/7 Activity Assay**

Cells (25,000 in 90 µL media) were plated in white-walled, clear-bottomed 96-well plates and allowed to adhere overnight. Cells were treated with compounds (10 µL, final 0.1% DMSO) in triplicate. After 21 hours, 10 µL alamarBlue was added and plates incubated for 3 hours. At 24 hours, fluorescence (Ex 560, Em 590) was measured to assess viability. After equilibration to room temperature for 15 min, media was removed and 110 µL Caspase-Glo 3/7 reagent added per well. Luminescence (1000 ms) was measured in kinetic mode every 3 minutes for 3 hours. Luminescence data at 45 minutes were normalized to alamarBlue data and fold changes reported relative to DMSO control.

**Cell Cycle Assay**

HCT116 (WT, *MAP3K1* KO, and *CRBN* KO) and HeLa cells (1 × 10⁶ in 4 mL media) were plated in T-25 flasks, allowed to adhere overnight, treated with compounds, and incubated for 24 hours. Cells were trypsinized, pelleted, and fixed in 70% ethanol at 4°C for 1 hour. After washing with PBS, pellets were resuspended in Telford Reagent (115 μM EDTA, 27 μg/mL RNAseA, 50 μg/mL propidium iodide, 0.1% Triton X-100 in 1X PBS) and incubated at 4°C for 1 hour or overnight. Flow cytometry was performed at the UNMC Flow Cytometry Core Facility (Calibur 3). The percentage of cells in G1, G2, and S phases was determined using ModFit LT™ software (n=3).

**Wound Healing/Migration Assay**

HCT116 WT and *MAP3K1* KO cells (150,000 in 90 µL media) were plated in 96-well plates. Scratches were scored with a 10 µL pipette tip, gently aspirated media, wells rinsed twice with 100 µL 1X PBS, and 90 µL fresh media added. Cells were treated with 5-fold dilutions of HBC-8 (in triplicate) starting at 10 µM in 0.1% DMSO. Plates were monitored in an Incucyte® at 10X wide field, scanning every hour for 24 hours.

**Live-Cell Apoptosis Assay**

T47D and NCIH1048 cells (40,000 in 100 µL media) were plated in black, clear-bottom 96-well plates and attached overnight. Media was replaced with 100 µL fresh RPMI containing 1.5 mM CaCl₂, 10 µL/mL pSIVA-IANBD, and 10 µL/mL propidium iodide (from Annexin V/PI Apoptosis Detection Kit). After 1 hour incubation at 37°C, cells were treated with 1 µM HBC-8 (in triplicate, final 0.1% DMSO) and monitored in an Incucyte® at 4X wide field every hour for 72 hours. Images were collected on three channels (phase, green, red) and processed using GraphPad Prism 10.5.0.

**Organoid Viability Assay**

Colon cancer organoid Q-20-70T was obtained from the Organoid Core of the Nebraska Center for Molecular Target Discovery and Development. Organoids were cultured in Advanced DMEM/F12 containing 20% R-Spondin conditioned medium, 10% Noggin conditioned medium, N21-Max supplement, 10 mM nicotinamide, 1.25 mM N-acetylcysteine, 100 μg/mL Primocin, 50 ng/mL EGF, 3 µM SB202190, 500 nM A83-01, 1 µM PEG2, and 10 nM Gastrin at 37°C in 5% CO_2_ (1). For treatments, organoids were cultured in suspension in 100 μL medium containing 5% BME in 96-well plates. Organoids were treated with vehicle or varying concentrations of HBC-2, HBC-8, HBC-8C (N-methylated thalidomide control), and HBC-23 (modified MAP3K1 ligand control) (6 wells per condition). After 72 hours, 50 μL of 0.2% MTT in PBS was added for 2 hours before formazan crystals were dissolved with 50 μL 20% SDS, 20 mM HCl. Absorbance at 570 nm was measured using a BioTek EL808 plate reader, and viability was calculated relative to vehicle-treated controls.

**High-Throughput PRISM Pooled Cell Sensitivity Screen**

HBC-8 test agent was treated at 8 doses in triplicate across 900 PRISM barcoded cancer cell lines for five days at the Broad Institute (2). All cell lines were cultured in RPMI 1640 with 10% FBS. Adherent cell lines were seeded at 1,250 cells/well, and suspension cell lines were seeded at 2000 cells/well in pools of 20-25 cell lines per pool. Pools of cell lines were lysed with Qiagen TCL buffer to isolate the mRNA. mRNA was then reverse transcribed into cDNA, and then the sequence containing the unique PRISM barcode was amplified using PCR. Finally, Luminex beads that recognize the reverse complement of the specific barcode sequences in the cell set were hybridized to the PCR products. The magnetic beads attached to the PCR products were then detected using a Luminex FlexMap 3D scanner, which reports a median fluorescent intensity (MFI) signal. The data was then processed to calculate AUC, IC_50_, and create dose-response curves. The details of the data processing steps are at: https://github.com/cmap/dockerized_mts

**Gene Dependency Analysis**

Gene dependency analysis used IC_50_ data from the PRISM screen. From post-Chronos gene dependency probability estimates (DEPMAP Gene Dependency 24Q4), a gene was labeled a “dependency” when its probability threshold was >0.5. Percent dependency was calculated as: (number of sensitive or resistant cell lines with gene dependency / total cell lines) × 100. Genes were ranked by maximum difference in percentage between groups to identify potential genetic mediators of sensitivity and resistance.

**Mass Spectrometry and Bioinformatics Analysis**

A total of 200ng of peptides were loaded onto the Vanquish Neo-uHPLC for mass spectrometry analysis. A trap-and-elute strategy using the PepMap Neo trap cartridge and μPAC Neo HPLC column. The column compartment was heated to 50°C. Samples were subjected to a 34-minute gradient, with Buffer A (99.9% H_2_O, 0.1% formic acid (FA)) and Buffer B (80% acetonitrile, 19.9% H_2_O, and 0.1% FA). The gradient was as follows: 4% Buffer B (0-0.4 min), 4-8% Buffer B (0.4-1.1 min), 8-22.5% Buffer B (1.1-16.1 min), 22.5-45% Buffer B (16.1-27.1 min), 45-99% Buffer B (27.1-27.6 min), and 99% Buffer B (27.6-34 min). MS analysis was performed using the Thermo Scientific Orbitrap-ASTRAL system. Instrument control and data acquisition were managed with the Xcalibur software, collecting data for MS1 and DIA analysis. For MS1 settings, Easy-IC was used for Orbitrap Lock Mass Correction; Orbitrap resolution was set to 240,000; scan range was 380–980 m/z; RF Lens was set to 40%; normalized AGC target was 300%; maximum injection time was 10 ms; polarity was positive with 1 micro scan. For DIA settings, Easy-IC was also used for Orbitrap Lock Mass Correction; precursor mass range was 380–980 m/z; DIA window type was set to auto; isolation window was 2 m/z, no window overlap, normalized collision energy type, HCD Collision energy of 25%; Astral was used as the detector type, a scan range of 150–2,000 m/z; RF Lens was set to 45%; normalized AGC target was 500%; and a maximum injection time was 3.5 ms.

Data files were analyzed using Spectronaut version 20.0 using the Direct DIA analysis function. The reviewed Swiss-Prot Human database from 01/01/2024, containing 20,428 protein entries, was used for analysis. Digest settings were set to Trypsin/P with Lys-c with peptide fragments of 7-52 amino acids, with a maximum of 2 missed cleavages. Pulsar search with modifications was done using a closed search with a maximum of 5 modifications; those include Acetylation (Protein N-term), Carbamidomethylation (C), and oxidation (M). DIA analysis Identification settings were changed to include a precursor Q-value cutoff of 0.001, a precursor PEP cutoff of 0.05, and a protein PEP cutoff of 0.1. Single hit definition is done by a stripped sequence, and the single hit protein rule of stratified single hit protein FDR. DIA analysis quantification settings were set to use the background signal for imputation, and the MS2 level was used for quantification. The mass spectrometry proteomics data have been deposited to the ProteomeXchange Consortium via the PRIDE (3) partner repository with the dataset identifier PXD070052. The reviewer access details are provided below.

Log in to the PRIDE website using the following details:

Project accession: PXD070052

Token: Mjpyg6imZBlI

Alternatively, the reviewer can access the dataset by logging in to the PRIDE website using the following account details:

Username: reviewer_pxd070052@ebi.ac.uk

Password: R0G60YADb3Xy

**Chemogenomic CRISPR Screen**

The Genome-wide pooled CRISPR/Cas9 KO screen was performed by the ChemoGenix platform (IRIC, Université de Montréal; https://chemogenix.iric.ca/) as previously described. Briefly, a NALM6 clone bearing an integrated inducible Cas9 expression cassette generated by lentiviruses made from pCW-Cas9 (Addgene #50661) was transduced with the genome-wide KO EKO sgRNA library (278,754 different sgRNAs) (PMID: 29038160). After thawing the library from liquid nitrogen and letting it recover in 10% FBS RPMI for 1 day, KOs were induced for 7 days of culture with 2 µg/mL doxycycline. The pooled library was split into T-75 flasks (28 x 10⁶ cells per flask; representing 100 cells/sgRNA) in 70 mL at 4 x 10⁵ cells/mL. Cells were treated with 0.15 µM of HBC-8 for 8 days, with monitoring of growth every 2 days, diluting back to 4 x 10⁵ cells/mL and adding more compound to maintain the same final concentration whenever cells reached 8 x 10⁵ cells/mL. Over that period, treated cells had 1.53 population doublings, whereas DMSO-only treated negative controls had 7.5. Cells were collected, genomic DNA extracted using the Gentra Puregene kit according to the manufacturer’s instructions, and sgRNA sequences PCR-amplified as described (PMID: 29038160). sgRNA frequencies were obtained by next-generation sequencing (Illumina NextSeq 2000). Reads were aligned using Bowtie 2.4.4 in the forward direction only (–norc option) with otherwise default parameters, and total read counts per sgRNA were tabulated. Context-dependent chemogenomic interaction scores were calculated using a modified version of the CRANKS algorithm (PMID: 29038160) that uses guides targeting similarly essential genes as controls to distinguish condition-specific chemogenomic interactions from non-specific fitness/essentiality phenotypes. Data is available in GEO accession number: GSE347763.

**WBC Colony Formation Assay**

Human CD34+ hematopoietic stem cells (2,000 cells/mL in 500 μL) were suspended in Methocult M3234 containing 10 ng/mL IL-3 and 1:1000 mSCF and plated in 12-well plates. Cells were treated with varying concentrations of HBC-8 and incubated at 37°C in 5% CO₂ for 14 days. Colonies were scored and categorized as CFU-E (Colony Forming Unit-Erythroid) or CFU-GM (Colony Forming Unit-Granulocyte/Macrophage). Data are presented as mean ± SEM.

**Animal Studies (reported per ARRIVE guidelines)**

**Animals**

Female athymic nude mice (stock no. 007850) aged 5–6 weeks were purchased from The Jackson Laboratory (Bar Harbor, ME, USA). Animals were purpose-bred by the supplier; no in-house breeding was carried out. On arrival, mice were acclimatized for one week before any procedure. Mice were housed in individually ventilated cages, five animals per cage, under specific pathogen-free conditions. Housing and husbandry were provided and monitored by UNMC Comparative Medicine in accordance with institutional standard operating procedures and the NIH Guide for the Care and Use of Laboratory Animals, with room temperature, relative humidity and light cycle maintained within the ranges specified therein (20–26 °C and 30–70% relative humidity). Food and water were available ad libitum. Animals were checked by veterinary staff twice weekly, with the frequency increased as required by clinical conditions. All procedures were reviewed and approved by the Institutional Animal Care and Use Committee of the University of Nebraska Medical Center (protocol no. 17-037-06) and conducted in accordance with the NIH Guide for the Care and Use of Laboratory Animals. Reporting follows the ARRIVE 2.0 guidelines.

**Tumor cell inoculation**

SKOV3-luciferase cells were harvested, washed in PBS and resuspended in PBS:Matrigel 1:1 (v/v) on ice. Each mouse received a single intraperitoneal injection of 5 × 10⁶ cells in 200 µL using a 1/2 mL BD Tuberculin syringe with a permanently attached 27 G × 1/2 in. needle.

**Randomization and group allocation**

Tumor establishment was monitored by bioluminescence imaging. When total flux reached approximately 1 × 10⁸ photons/s (ventral and dorsal regions of interest), designated day 0, mice were allocated to treatment groups. Group sizes were n = 5 (vehicle), n = 4 (HBC-8 7.5 mg/kg) and n = 5 (HBC-8 15 mg/kg), giving 14 animals in total. Sample size was based on our previous experience with this model rather than a formal power calculation. Animals were identified by ear tag.

**Treatment**

From day 0, mice received vehicles (5% DMSO, 40% PEG-400, 10% Cremophor, 45% Tris buffer [10 mM, pH 8.0, 150 mM NaCl]) or HBC-8 at 7.5 or 15 mg/kg by intraperitoneal injection, six days per week for four weeks. Injection volume was adjusted to body weight and ranged from 100 to 150 µL per animal. Investigators were not blinded to group allocation during treatment administration or imaging.

**Tumor burden measurement**

Tumor burden was assessed weekly by bioluminescence imaging. Mice received D-luciferin intraperitoneally at 10 µL/g from a 15 mg/mL stock (approximately 150 mg/kg) and were anaesthetized with isoflurane (2–3% for induction, 1.5–2% for maintenance) in oxygen. Images were acquired 10-15 min after substrate injection on an IVIS Spectrum system using auto exposure, small binning, f/stop 1 and field of view D. Total flux (photons/s) and average radiance (photons/s/cm²/sr) were quantified within ventral and dorsal regions of interest using Living Image v4.8.2, with identical ROI dimensions applied across all animals and timepoints. Body weight was recorded twice weekly as a tolerability measure.

**Humane endpoints, exclusions and euthanasia**

Animals were monitored for body weight loss, abdominal distension, reduced mobility, hunching and coat condition. Predefined humane endpoints were carried out as per the approved criteria from protocol 17-037-06 at which point animals were euthanized early. Two vehicle-treated animals reached a humane endpoint before day 28 and were euthanized at that time; their tissues were collected and included in the endpoint analyses. No animals were excluded from analysis, and no treatment-related adverse events were observed in either HBC-8 group. Mice were euthanized at the study endpoint on day 28, or earlier if a humane endpoint was reached, by CO₂ inhalation delivered at a displacement rate of 30–70% of chamber volume per minute, followed by cervical dislocation as a secondary physical method, in accordance with the AVMA Guidelines for the Euthanasia of Animals (2020). Death was confirmed by absence of heartbeat and respiration before tissue collection. Immediately after euthanasia, peritoneal organs were excised for ex vivo bioluminescence imaging and tumor nodules were collected for histology.

**Immunohistochemistry**

Immunohistochemistry was performed using the previously reported method (4).

**Key Resources Table**

| **Reagent / Resource** | **Source** | **Identifier** |
| --- | --- | --- |
| **Antibodies** | | |
| anti-MAP3K1 | Bethyl | A302-395A |
| anti-pERK1/2 | Cell Signaling Technology (CST) | 9106S |
| anti-ERK1/2 | BD Biosciences | 610031 |
| anti-pIKKα/β | CST | 2078S |
| anti-IKKα/β | CST | 2684S |
| anti-pJNK | CST | 4668 |
| anti-JNK | CST | 9252 |
| anti-DUSP4 | CST | 5149 |
| anti-pAKT (Thr308) | CST | 13038 |
| anti-pAKT (Ser473) | CST | 4060 |
| anti-AKT | CST | 9272 |
| anti-pMEK1/2 | Thermo Fisher | 44-454G |
| anti-MEK | CST | 8727 |
| anti-CRBN | CST | D8H3S |
| anti-Vinculin | CST | 13901 |
| anti-α-tubulin | CST | 2146S |
| anti-HSP90 | CST | 4874 |
| anti-CDK4 | CST | 12790S |
| anti-CDK2 | CST | 2546S |
| anti-CDK12 | CST | 45791 |
| anti-CDK1 | CST | 77055 |
| anti-CDK9 | CST | 2316 |
| anti-MAP2K6 | CST | 8550 |
| anti-p38 | CST | 8690 |
| anti-ASK1 | CST | 8662 |
| anti-FAK | CST | 13009 |
| anti-DLK1 | CST | 94845 |
| anti-TRAF1 | CST | 4715 |
| anti-AMPK | CST | 5831 |
| anti-CUL4A (IP) | CST | 2699S |
| anti-DDB1 (IP) | CST | 5428S |
| HRP anti-rabbit IgG (2°) | Invitrogen | 32460 |
| HRP anti-mouse IgG (2°) | Invitrogen | G21040 |
| Normal IgG (IP control) | CST | 2729S |
| Precision Plus protein ladder | Bio-Rad | 1610375 |
| **Chemicals, peptides & recombinant proteins** | | |
| HBC-1 – HBC-23 (this study) | This study | — |
| 51-106 (iMAP3K1) / IKAM-1 warheads | Cited in previous publications | — |
| Pomalidomide | Selleck Chemicals | P0018 |
| DMSO | Fisher Scientific | BP231 |
| BSA | Fisher Bioreagents | BP9706 |
| Tris-Glycine-SDS buffer | Research Products Intl. | T32080 |
| MG-132 | Selleck Chemicals | 133407-82-6 |
| MLN-4924 (pevonedistat) | Med Chem Express | 905579-51-3 |
| Etoposide | Selleck Chemicals | 33419-42-0 |
| Z-VAD-FMK | Selleck Chemicals | 187389-52-2 |
| Doxycycline | Millipore Sigma | 10592-13-9 |
| Matrigel (PathClear) | Corning / PathClear | 36-320-0502 |
| D-luciferin | Syd Labs | 115144-35-9 |
| RIPA lysis buffer | Thermo Scientific | 89900 |
| MTT | Tocris | 5224500 |
| Insulin | Sigma-Aldrich | 11070-73-8 |
| L-glutamine | Gibco | 25030-081 |
| Propidium iodide | Invitrogen | P3566 |
| RNase A | Thermo Scientifi | R1253 |
| Protein A/G beads | Thermo Scientific | 20422 |
| PVDF membrane | Sigma aldrich | IPVH00010 |
| **Commercial assays / kits** | | |
| PrestoBlue Cell Viability Reagent | Invitrogen | A13262 |
| alamarBlue | Bio-Rad | BUF012B |
| Caspase-Glo 3/7 Assay | Promega | G8093 |
| Annexin V/PI (pSIVA-IANBD) Apoptosis Detection Kit | Abcam | ab129817 |
| Pierce BCA Protein Assay Kit | Thermo Scientific | 23225 |
| ECL Prime | GE Healthcare (Cytiva) | RPN2236 |
| AccelerOme Label-Free MS Sample Prep Kit | Thermo | A50945 |
| Pierce Quantitative Peptide Assay Kit | Thermo | 23290 |
| MethoCult M3234 | STEMCELL Technologies | M3234 |
| **Cell lines** | | |
| HCT116 wild-type | ATCC | CCL-247 |
| HCT116 *MAP3K1* KO | MSKCC Genome Editing Core | (5) |
| HCT116 *CRBN* KO | UNMC Genome Editing Core | This study |
| T47D | V. Band Lab, UNMC | — |
| NCIH1048 | ATCC | CRL-5853 |
| SKOV3-luciferase | Jixin Dong Lab, UNMC | — |
| HeLa | ATCC | CRM-CCL-2 |
| NALM6 (inducible Cas9) | IRIC ChemoGenix | — |
| Colon-cancer organoid Q-20-70T | Organoid Core of the Nebraska Center for Molecular Target Discovery and Development | — |
| **Media & culture reagents** | | |
| McCoy’s 5A medium | Cytiva | SH30200.01 |
| RPMI 1640 | Cytiva | SH30027.01 |
| DMEM/F12 | Cytiva | SH30261.01 |
| Fetal bovine serum (FBS) | Cytiva | 35-015-CV |
| Penicillin-Streptomycin | Cytiva | SV30010 |
| PBS | Cytiva | SH30256.01 |
| **Experimental models: organisms** | | |
| Female athymic nude mice, 5–6 wk | The Jackson Laboratory | 007850 |
| **Oligonucleotides, libraries & constructs** | | |
| Genome-wide EKO sgRNA library (278,754 sgRNAs) | IRIC ChemoGenix | PMID: 29038160 |
| pCW-Cas9 | Addgene | 50661 |
| **Instruments** | | |
| Orbitrap Astral MS | Thermo Scientific | — |
| Vanquish Neo UHPLC | Thermo Scientific | — |
| PepMap Neo trap cartridge | Thermo | 174500 |
| µPAC Neo HPLC column | Thermo | COL-NANO050NEOB |
| ChemiDoc imaging system | Bio-Rad | — |
| SpectraMax M5e plate reader | Molecular Devices | — |
| BioTek EL808 plate reader | BioTek | — |
| Incucyte live-cell imager | Sartorius | — |
| FACSCalibur flow cytometer | BD Biosciences | — |
| Illumina NextSeq 2000 sequencer | Illumina | — |
| SCIEX QTRAP 4000 (LC-MS/MS) | SCIEX | — |
| IVIS Spectrum in vivo imaging | PerkinElmer | — |
| **Software & deposited data** | | |
| CRANKS algorithm (modified) | — | PMID: 29038160 |
| GraphPad Prism 10.5.0 | GraphPad | — |
| Spectronaut 20.0 | Biognosys | — |
| Xcalibur | Thermo Scientific | — |
| Bowtie 2.4.4 | open source | — |
| ModFit LT | Verity Software House | — |
| Living Image v4.8.2 | PerkinElmer | — |
| WinNonlin 1.5 | Certara | — |
| ImageJ | NIH | — |
| Proteomics raw data | ProteomeXchange / PRIDE | PXD070052 |

**Chemistry Supplementary Information.**

**Heterobifunctional Compounds (HBC) Synthesis**

**Methyl 4-amino-2-(trifluoromethyl)benzoate (2)**

To a stirred solution of methyl 4-nitro-2-(trifluoromethyl)benzoate (1) (200 mg, 0.802 mmol) in EtOH was added 5% Pd/C (20 mg). The mixture was stirred under a hydrogen atmosphere for 12 hours. After the completion of the reaction, the reaction mixture was filtered over Celite and evaporated. Yield = 95%; **^1^H NMR** (400 MHz, CDCl_3_) δ 7.76 (d, *J* = 8.4 Hz, 1H), 6.98 (d, *J* = 2.4 Hz, 1H), 6.76 (dd, *J* = 8.4, 2.5 Hz, 1H), 4.26 – 4.15 (m, 2H), 3.87 (s, 3H).

**Methyl 4-isocyanato-2-(trifluoromethyl)benzoate (3)**

To a stirred solution of triphosgene (75 mg, 0.252 mmol) in toluene (8 mL) was added a solution of 2 (166.12 mg, 0.758 mmol) in dichloromethane (4 mL) at 0 °C. The mixture was stirred for 30 minutes and refluxed for 10 hours. The solvent was removed under reduced pressure, and the crude was used as such for the next reaction; Yield = 65%.

**Methyl4-(3-(3-(1-methyl-1H-pyrazol-4-yl)quinoxalin-6-yl)ureido)-2-(trifluoromethyl)benzoate (5a)**

To a solution of 2 (25 mg, 0.110 mmol) in dichloromethane (4 mL) was slowly added a solution of 51-112 (81.62 mg, 0.332 mmol) in dichloromethane (1 mL). The solution was stirred for 2 days. After completion of the reaction, the solvent was removed under reduced pressure and purified using column chromatography; Yield = 68%; **^1^H NMR** (500 MHz, DMSO) δ 9.62 (s, 1H), 9.49 (s, 1H), 9.13 (s, 1H), 8.62 (s, 1H), 8.31 – 8.23 (m, 2H), 8.16 (d, *J* = 2.1 Hz, 1H), 7.95 (d, *J* = 9.0 Hz, 1H), 7.89 (d, *J* = 8.5 Hz, 1H), 7.78 (dd, *J* = 8.7, 2.1 Hz, 1H), 7.71 (dd, *J* = 9.1, 2.4 Hz, 1H), 3.95 (s, 3H), 3.85 (s, 3H).**^13^C NMR** (126 MHz, DMSO) δ 165.8, 152.2, 147.4, 143.0, 142.6, 141.7, 140.7, 138.0, 136.9, 132.2, 131.0, 129.3, 128.5, 128.3,123.3 (q, *J =* 273.123 Hz),122.7, 121.9, 120.8, 120.2, 115.7, 113.9, 52.6, 38.9.**^19^F NMR** (376 MHz, DMSO) δ -58.57.

**4-(3-(3-(1-methyl-1H-pyrazol-4-yl)quinoxalin-6-yl)ureido)-2-(trifluoromethyl)benzoic acid (5b)**

To a solution of 5a (20 mg, 0.042 mmol) in Methanol (1 mL) and THF (1 mL) was added 1 mL of 1M NaOH. The mixture was stirred for 24 hours at 50 °C. After completion of the reaction, the solvent was removed under reduced pressure, and 4 mL of water was added to the mixture. 1N HCl was added to the clear solution until the pH reached 6. The precipitates formed were filtered and dried. Yield = 44%; **^1^H NMR** (500 MHz, DMSO) δ 13.26 (s, 1H), 9.85 (s, 1H), 9.75 (s, 1H), 9.14 (s, 1H), 8.63 (s, 1H), 8.28 (s, 1H), 8.26 (d, *J* = 2.4 Hz, 1H), 8.13 (d, *J* = 2.1 Hz, 1H), 7.95 (d, *J* = 8.9 Hz, 1H), 7.88 (d, *J* = 8.5 Hz, 1H), 7.74 (dd, *J* = 8.5, 2.2 Hz, 1H), 7.71 (dd, *J* = 9.0, 2.4 Hz, 1H), 3.95 (s, 3H).

**General procedure for the synthesis of HBC 1-23:**

To a solution of 5b (1.0 eq) and amine with E3-ligase ligand (1.0 eq) in DMF (1 mL), DIPEA (4.0 eq) was added and stirred for 10 minutes, followed by the addition of HATU (2.0 eq). The reaction mixture was stirred at room temperature for 16h. The crude was purified by reverse-phase HPLC (10-100% CH_3_CN in H_2_O) and lyophilized to give the product as fluorescent solids. Yields: 30-80%.

**HBC-1**

**(N-(14-((2-(2,6-dioxopiperidin-3-yl)-1,3-dioxoisoindolin-4-yl)amino)-3,6,9,12-tetraoxatetradecyl)-4-(3-(3-(1-methyl-1H-pyrazol-4-yl)quinoxalin-6-yl)ureido)-2-(trifluoromethyl)benzamide)**

**^1^H NMR** (500 MHz, MeOD) δ 9.02 (s, 1H), 8.45 (s, 1H), 8.30 – 8.23 (m, 2H), 7.98 – 7.89 (m, 2H), 7.76 (ddd, *J =* 31.5, 8.8, 2.4 Hz, 2H), 7.54 – 7.42 (m, 2H), 6.99 (dd, *J =* 15.2, 7.8 Hz, 2H), 5.03 (dd, *J =* 12.6, 5.5 Hz, 1H), 4.01 (s, 3H), 3.69 (t, *J =* 5.3 Hz, 2H), 3.62 (p, *J =* 4.7 Hz, 14H), 3.53 (t, *J =* 5.3 Hz, 2H), 3.45 (t, *J =* 5.4 Hz, 2H), 2.88 – 2.78 (m, 1H), 2.77 – 2.67 (m, 2H), 2.10 (dtd, *J =* 13.1, 5.7, 2.7 Hz, 1H). **HRMS** **(ESI-MS)** calcd for C_44_H_46_F_3_N_10_O_10_^+^ m/z (M+H)^+^ 931.3345, found: 931.3340.

**HBC-2**

**(N-(2-((2-(2,6-dioxopiperidin-3-yl)-1,3-dioxoisoindolin-4-yl)amino)ethyl)-4-(3-(3-(1-methyl-1H-pyrazol-4-yl)quinoxalin-6-yl)ureido)-2-(trifluoromethyl)benzamide)**

**^1^H NMR** (400 MHz, DMSO-*d*_6_) δ 11.10 (s, 1H), 9.54 (d, *J =* 8.8 Hz, 1H), 9.13 (s, 1H), 8.61 (s, 1H), 8.31 (s, 1H), 8.28 – 8.24 (m, 2H), 8.05 (d, *J =* 2.3 Hz, 1H), 7.95 (d, *J =* 8.9 Hz, 1H), 7.70 (td, *J =* 8.5, 2.4 Hz, 2H), 7.63 (dd, *J =* 8.6, 7.0 Hz, 1H), 7.47 (d, *J =* 8.5 Hz, 1H), 7.24 (d, *J =* 8.6 Hz, 1H), 7.06 (d, *J =* 7.0 Hz, 1H), 6.81 (s, 1H), 5.08 (dd, *J =* 12.9, 5.7 Hz, 1H), 3.95 (s, 3H), 3.43 (d, *J =* 4.9 Hz, 4H), 2.96 – 2.84 (m, 1H), 2.64 – 2.52 (m, 2H), 2.11 – 1.96 (m, 1H). **^13^C NMR** (126 MHz, DMSO-*d*_6_) δ 172.9, 172.8, 170.1, 168.7, 167.4, 152.4, 152.4, 147.4, 146.3, 142.6, 141.6, 140.9, 140.8, 138.0, 136.8, 136.3, 132.3, 131.0, 129.6, 129.3, 126.8 (q, *J =* 31.9 Hz), 123.6 (q, *J =* 275.1 Hz), 121.9, 121.0, 120.2, 117.2, 115.3, 113.6, 110.7, 109.4, 48.6, 41.0, 38.5, 31.0, 22.2. **^19^F NMR** (376 MHz, DMSO-*d*_6_) δ -58.12. **HRMS (ESI-MS)** calcd for C_36_H_30_F_3_N_10_O_6_^+^ m/z (M+H)^+^ 755.2296, found: 755.2296.

**HBC-3**

**(N-(2-(2-(2-((2-(2,6-dioxopiperidin-3-yl)-1,3-dioxoisoindolin-4-yl)amino)ethoxy)ethoxy)ethyl)-4-(3-(3-(1-methyl-1H-pyrazol-4-yl)quinoxalin-6-yl)ureido)-2-(trifluoromethyl)benzamide)**

**^1^H NMR** (499 MHz, MeOD) δ 8.90 (s, 1H), 8.33 (s, 1H), 8.18 – 8.13 (m, 2H), 7.85 – 7.77 (m, 2H), 7.62 (dd, *J =* 9.0, 2.4 Hz, 1H), 7.55 (dd, *J =* 8.4, 2.1 Hz, 1H), 7.40 (dd, *J =* 8.6, 7.1 Hz, 1H), 7.34 (d, *J =* 8.4 Hz, 1H), 6.91 (dd, *J =* 7.8, 3.9 Hz, 2H), 3.88 (s, 3H), 3.60 (t, *J =* 5.3 Hz, 2H), 3.57 – 3.54 (m, 4H), 3.41 (t, *J =* 5.4 Hz, 2H), 3.33 (d, *J =* 5.3 Hz, 2H), 2.76 – 2.65 (m, 2H), 2.63 – 2.53 (m, 2H), 2.03 – 1.89 (m, 2H). **HRMS** **(ESI-MS)** calcd for C_40_H_38_F_3_N_10_O_8_^+^ m/z (M+H)^+^ 843.2821, found: 843.2820.

**HBC-4**

**(N-(5-((2-(2,6-dioxopiperidin-3-yl)-1,3-dioxoisoindolin-4-yl)amino)pentyl)-4-(3-(3-(1-methyl-1H-pyrazol-4-yl)quinoxalin-6-yl)ureido)-2-(trifluoromethyl)benzamide)**

**^1^H NMR** (499 MHz, MeOD) δ 9.03 (s, 1H), 8.46 (s, 1H), 8.32 – 8.25 (m, 2H), 7.94 (dd, *J =* 5.6, 3.3 Hz, 2H), 7.76 (ddd, *J =* 14.0, 8.7, 2.3 Hz, 2H), 7.55 (dd, *J =* 8.6, 7.1 Hz, 1H), 7.44 (d, *J =* 8.5 Hz, 1H), 7.04 (dd, *J =* 13.5, 7.8 Hz, 2H), 5.06 (dd, *J =* 12.5, 5.5 Hz, 1H), 4.01 (s, 3H), 3.37 (q, *J =* 6.8 Hz, 4H), 2.90 – 2.82 (m, 1H), 2.79 – 2.66 (m, 2H), 2.15 – 2.08 (m, 1H), 1.70 (dq, *J =* 27.2, 7.1 Hz, 4H), 1.60 – 1.49 (m, 2H). **HRMS** **(ESI-MS)** calcd for C_39_H_36_F_3_N_10_O_6_^+^ m/z (M+H)^+^ 797.2766, found: 797.2751.

**HBC-5**

**(N-(8-((2-(2,6-dioxopiperidin-3-yl)-1,3-dioxoisoindolin-4-yl)amino)octyl)-4-(3-(3-(1-methyl-1H-pyrazol-4-yl)quinoxalin-6-yl)ureido)-2-(trifluoromethyl)benzamide)**

**^1^H NMR** (499 MHz, MeOD) δ 9.04 (s, 1H), 8.47 (s, 1H), 8.32 – 8.27 (m, 2H), 8.00 – 7.93 (m, 2H), 7.76 (dt, *J =* 9.0, 2.5 Hz, 2H), 7.55 (dd, *J =* 8.6, 7.1 Hz, 1H), 7.46 (d, *J =* 8.2 Hz, 1H), 7.04 (dd, *J =* 7.8, 4.4 Hz, 2H), 5.05 (dd, *J =* 12.6, 5.5 Hz, 1H), 4.02 (s, 3H), 3.39 – 3.32 (m, 4H), 2.85 (ddd, *J =* 17.8, 14.2, 5.3 Hz, 1H), 2.78 – 2.68 (m, 2H), 2.10 (ddd, *J =* 12.9, 5.2, 2.7 Hz, 1H), 1.73 – 1.53 (m, 4H), 1.52 – 1.32 (m, 8H). **HRMS (ESI-MS)** calcd for C_42_H_42_F_3_N_10_O_6_^+^ m/z (M+H)^+^ 839.3235, found: 839.3231.

**HBC-6**

**(N-(7-((2-(2,6-dioxopiperidin-3-yl)-1,3-dioxoisoindolin-4-yl)amino)heptyl)-4-(3-(3-(1-methyl-1H-pyrazol-4-yl)quinoxalin-6-yl)ureido)-2-(trifluoromethyl)benzamide)**

**^1^H NMR** (499 MHz, MeOD) δ 9.08 (s, 1H), 8.50 (s, 1H), 8.34 (d, *J =* 2.3 Hz, 1H), 8.31 (s, 1H), 8.02 – 7.96 (m, 2H), 7.78 (dd, *J =* 9.0, 2.3 Hz, 2H), 7.56 (dd, *J =* 8.6, 7.1 Hz, 1H), 7.47 (d, *J =* 8.3 Hz, 1H), 7.08 – 7.02 (m, 2H), 5.09 – 5.05 (m, 1H), 4.04 (s, 3H), 3.40 – 3.35 (m, 4H), 2.90 – 2.84 (m, 1H), 2.78 – 2.71 (m, 2H), 2.15 – 2.09 (m, 1H), 1.72 (t, *J =* 7.1 Hz, 2H), 1.67 – 1.62 (m, 2H), 1.47 (d, *J =* 4.5 Hz, 6H). **HRMS** **(ESI-MS)** calcd for C_41_H_40_F_3_N_10_O_6_^+^ m/z (M+H)^+^ 825.3079, found: 825.3084.

**HBC-7**

**(N-(2-(2-(2-(2-((2-(2,6-dioxopiperidin-3-yl)-1,3-dioxoisoindolin-4-yl)amino)ethoxy)ethoxy)ethoxy)ethyl)-4-(3-(3-(1-methyl-1H-pyrazol-4-yl)quinoxalin-6-yl)ureido)-2-(trifluoromethyl)benzamide)**

**^1^H NMR** (499 MHz, MeOD) δ 9.06 (s, 1H), 8.48 (s, 1H), 8.36 – 8.26 (m, 2H), 7.99 – 7.92 (m, 2H), 7.77 (ddd, *J =* 15.7, 8.7, 2.3 Hz, 2H), 7.56 – 7.48 (m, 2H), 7.06 (d, *J =* 8.5 Hz, 1H), 7.01 (d, *J =* 7.0 Hz, 1H), 5.05 (dd, *J =* 12.6, 5.5 Hz, 1H), 4.04 (s, 3H), 3.79 – 3.58 (m, 10H), 3.55 (t, *J =* 5.3 Hz, 2H), 3.48 (t, *J =* 5.2 Hz, 2H), 2.95 – 2.77 (m, 2H), 2.80 – 2.67 (m, 3H), 2.16 – 2.05 (m, 1H). **HRMS (ESI-MS)** calcd for C_42_H_42_F_3_N_10_O_9_^+^ m/z (M+H)^+^ 887.3083, found: 887.3087.

**HBC-8**

**N-(3-((2-(2,6-dioxopiperidin-3-yl)-1,3-dioxoisoindolin-4-yl)amino)propyl)-4-(3-(3-(1-methyl-1H-pyrazol-4-yl)quinoxalin-6-yl)ureido)-2-(trifluoromethyl)benzamide**

**^1^H NMR** (499 MHz, Methanol-d4) δ 9.06 (s, 1H), 8.49 (s, 1H), 8.34 – 8.28 (m, 2H), 8.01 – 7.95 (m, 2H), 7.79 (td, *J =* 9.2, 2.3 Hz, 2H), 7.59 (dd, *J =* 8.6, 7.1 Hz, 1H), 7.51 (d, *J =* 8.4 Hz, 1H), 7.10 (dd, *J =* 18.7, 7.8 Hz, 2H), 5.08 (dd, *J =* 12.4, 5.5 Hz, 1H), 4.03 (s, 3H), 3.50 (dt, *J =* 11.7, 6.7 Hz, 4H), 2.89 (ddd, *J =* 17.9, 14.1, 5.2 Hz, 1H), 2.82 – 2.71 (m, 2H), 2.18 – 2.10 (m, 1H), 2.03 – 1.93 (m, 2H). **^13^C NMR** δ 172.8, 169.8, 169.0, 168.8, 167.4, 152.3, 146.8, 146.2, 142.1, 140.8, 140.7, 140.4, 137.6, 136.6, 135.3, 132.1, 130.4, 128.7, 128.2, 121.8, 120.6, 119.8, 116.1, 115.4, 113.3, 110.0, 109.4, 48.3, 39.2, 37.4, 36.7, 30.3, 28.0, 21.9. **^19^F NMR** (377 MHz, DMSO) δ -58.08. **HRMS (ESI-MS)** calcd for C_37_H_32_F_3_N_10_O_6_^+^ m/z (M+H)^+^ 769.2453, found: 769.2456.

**HBC-8c**

**4-(3-(3-(1-methyl-1H-pyrazol-4-yl)quinoxalin-6-yl)ureido)-N-(3-((2-(1-methyl-2,6-dioxopiperidin-3-yl)-1,3-dioxoisoindolin-4-yl)amino)propyl)-2-(trifluoromethyl)benzamide**

**^1^H NMR** (500 MHz, DMSO) δ 9.41 (d, *J =* 4.9 Hz, 2H), 9.13 (s, 1H), 8.62 (s, 1H), 8.52 (t, *J =* 5.7 Hz, 1H), 8.30 – 8.22 (m, 2H), 8.05 (d, *J =* 2.1 Hz, 1H), 7.95 (d, *J =* 9.0 Hz, 1H), 7.71 (dd, *J =* 9.0, 2.3 Hz, 2H), 7.60 (dd, *J =* 8.6, 7.0 Hz, 1H), 7.51 (d, *J =* 8.4 Hz, 1H), 7.14 (d, *J =* 8.6 Hz, 1H), 7.04 (d, *J =* 7.0 Hz, 1H), 6.75 (t, *J =* 6.3 Hz, 1H), 5.13 (dd, *J =* 13.0, 5.4 Hz, 1H), 3.95 (s, 3H), 3.44 – 3.36 (m, 2H), 3.29 (d, *J =* 6.2 Hz, 2H), 3.02 (s, 3H), 3.00 – 2.89 (m, 1H), 2.80 – 2.71 (m, 1H), 2.56 (dd, *J =* 13.2, 4.4 Hz, 1H), 2.09 – 2.01 (m, 1H), 1.78 (t, *J =* 6.7 Hz, 2H). **^13^C NMR** (126 MHz, DMSO) δ 171.8, 169.9, 168.8, 167.3, 167.0, 152.4, 147.4, 146.3, 142.7, 141.6, 140.9, 140.6, 138.0, 136.8, 136.3, 132.3, 131.0, 129.6, 129.3, 123.7 (q, *J*=273.10 Hz), 121.9, 121.2, 120.2, 117.2, 115.4, 113.7, 110.5, 109.1, 49.1, 38.9, 36.5, 31.1, 28.5, 26.6, 21.4. **^19^F NMR** (377 MHz, DMSO) δ -58.09. **HRMS (ESI-MS)** calcd for C_38_H_34_F_3_N_10_O_6_^+^ m/z (M+H)^+^ 783.2609, found: 783.2911.

**HBC-9**

**(N-(2-(2-(2-((2-(2,6-dioxopiperidin-3-yl)-1-oxoisoindolin-4-yl)amino)ethoxy)ethoxy)ethyl)-4-(3-(3-(1-methyl-1H-pyrazol-4-yl)quinoxalin-6-yl)ureido)-2-(trifluoromethyl)benzamide)**

Yield =72%; **^1^H NMR** (499 MHz, MeOD) δ 9.05 (s, 1H), 8.47 (s, 1H), 8.33 – 8.26 (m, 2H), 8.00 – 7.88 (m, 2H), 7.76 (tt, *J =* 7.2, 2.0 Hz, 1H), 7.68 (dd, *J =* 8.4, 2.2 Hz, 1H), 7.47 (d, *J =* 8.4 Hz, 1H), 7.34 (t, *J =* 7.8 Hz, 1H), 7.12 (d, *J =* 7.4 Hz, 1H), 6.88 (d, *J =* 7.9 Hz, 1H), 5.13 (dd, *J =* 13.3, 5.2 Hz, 1H), 4.28 (d, *J =* 3.6 Hz, 1H), 4.02 (s, 3H), 3.72 (t, *J =* 5.5 Hz, 2H), 3.67 (d, *J =* 11.5 Hz, 5H), 3.55 (dq, *J =* 15.4, 4.7 Hz, 2H), 3.40 (t, *J =* 5.5 Hz, 2H), 3.35 (s, 2H), 2.88 (ddd, *J =* 18.6, 13.2, 5.4 Hz, 1H), 2.83 – 2.72 (m, 1H), 2.45 (qd, *J =* 13.2, 4.6 Hz, 1H), 2.24 – 2.09 (m, 1H). **HRMS (ESI-MS)** calcd for C_40_H_40_F_3_N_10_O_7_^+^ m/z (M+H)^+^ 829.3028, found: 829.3023.

**HBC-10**

**(N-(2-(2-((2-(2,6-dioxopiperidin-3-yl)-1-oxoisoindolin-4-yl)amino)ethoxy)ethyl)-4-(3-(3-(1-methyl-1H-pyrazol-4-yl)quinoxalin-6-yl)ureido)-2-(trifluoromethyl)benzamide)** Yield = 69%; **^1^H NMR** (499 MHz, MeOD) δ 9.05 (s, 1H), 8.47 (s, 1H), 8.35 – 8.24 (m, 2H), 8.02 – 7.91 (m, 2H), 7.76 (dd, *J =* 9.0, 2.4 Hz, 2H), 7.62 (dd, *J =* 8.3, 2.2 Hz, 1H), 7.53 – 7.20 (m, 2H), 7.09 (d, *J =* 7.5 Hz, 1H), 6.92 (d, *J =* 8.1 Hz, 1H), 5.10 (dd, *J =* 13.3, 5.2 Hz, 1H), 4.22 (d, *J =* 4.1 Hz, 1H), 4.02 (s, 3H), 3.76 (t, *J =* 5.3 Hz, 1H), 3.67 (t, *J =* 5.3 Hz, 2H), 3.56 (dd, *J =* 6.0, 4.3 Hz, 2H), 3.50 – 3.40 (m, 2H), 2.89 (ddd, *J =* 19.0, 13.5, 5.7 Hz, 1H), 2.83 – 2.72 (m, 1H), 2.66 (s, 1H), 2.31 (qd, *J =* 13.3, 4.6 Hz, 1H), 2.19 – 2.06 (m, 1H). **^13^C NMR** (176 MHz, DMSO) δ 172.9, 171.2, 168.8, 167.1, 152.4, 147.4, 143.6, 142.6, 141.6, 140.9, 140.6, 138.0, 136.8, 132.1, 131.0, 129.9, 129.5, 129.3, 129.2, 126.7 (q, *J*=31.58 Hz), 126.5, 123.6 (q, *J*=273.31 Hz), 121.9, 121.1, 120.2, 116.7, 113.7, 112.0, 110.3, 68.8, 68.6, 51.5, 45.7, 42.6, 40.0, 38.9, 31.2, 22.7. **^19^F NMR** (377 MHz, DMSO) δ -58.12, -74.59. **HRMS (ESI-MS)** calcd for C_38_H_36_F_3_N_10_O_6_^+^ m/z (M+H)^+^ 785.2766, found: 785.2760.

**HBC-11**

**(N-(2-(2-((2-(2,6-dioxopiperidin-3-yl)-1,3-dioxoisoindolin-4-yl)amino)ethoxy)ethyl)-4-(3-(3-(1-methyl-1H-pyrazol-4-yl)quinoxalin-6-yl)ureido)-2-(trifluoromethyl)benzamide)**

Yield = 70%; **^1^H NMR** (499 MHz, MeOD) δ 9.04 (s, 1H), 8.47 (s, 1H), 8.31 (d, *J =* 2.4 Hz, 1H), 8.30 – 8.26 (m, 1H), 7.95 (d, *J =* 9.1 Hz, 1H), 7.91 (d, *J =* 2.2 Hz, 1H), 7.75 (dt, *J =* 9.1, 2.6 Hz, 2H), 7.52 (dd, *J =* 8.7, 7.0 Hz, 1H), 7.45 (d, *J =* 8.4 Hz, 1H), 7.11 (d, *J =* 8.6 Hz, 1H), 7.00 (d, *J =* 7.0 Hz, 1H), 5.01 (dd, *J =* 12.8, 5.5 Hz, 1H), 4.01 (s, 3H), 3.75 (t, *J =* 5.2 Hz, 2H), 3.69 (t, *J =* 5.3 Hz, 2H), 3.56 (td, *J =* 5.1, 2.4 Hz, 2H), 3.53 (td, *J =* 4.9, 1.7 Hz, 2H), 3.33 (d, *J =* 15.4 Hz, 2H), 2.86 (ddd, *J =* 17.3, 13.9, 5.3 Hz, 1H), 2.76 – 2.62 (m, 2H), 2.11 (ddq, *J =* 11.1, 5.4, 2.7 Hz, 1H). **HRMS (ESI-MS)** calcd for C_38_H_34_F_3_N_10_O_7_^+^ m/z (M+H)^+^ 799.2559, found: 799.2553.

**HBC-12**

**(N-(1-((2-(2,6-dioxopiperidin-3-yl)-1,3-dioxoisoindolin-4-yl)oxy)-2-oxo-6,9,12-trioxa-3-azatetradecan-14-yl)-4-(3-(3-(1-methyl-1H-pyrazol-4-yl)quinoxalin-6-yl)ureido)-2-(trifluoromethyl)benzamide)**

Yield = 55%; **^1^H NMR** (499 MHz, MeOD) δ 9.02 (s, 1H), 8.46 (d, *J =* 3.8 Hz, 1H), 7.97 – 7.89 (m, 2H), 7.79 (dd, *J =* 8.4, 2.3 Hz, 1H), 7.73 – 7.66 (m, 2H), 7.51 (d, *J =* 8.4 Hz, 1H), 7.43 (d, *J =* 7.2 Hz, 1H), 7.32 (d, *J =* 8.4 Hz, 1H), 5.10 (dd, *J =* 12.7, 5.5 Hz, 1H), 4.71 (s, 2H), 4.01 (s, 3H), 3.68 – 3.61 (m, 9H), 3.59 (t, *J =* 5.4 Hz, 2H), 3.53 (t, *J =* 5.3 Hz, 2H), 3.47 (t, *J =* 5.3 Hz, 2H), 3.35 (s, 2H), 2.92 – 2.82 (m, 2H), 2.79 – 2.68 (m, 2H), 2.66 (s, 1H), 2.14 (ddd, *J =* 9.8, 5.1, 2.8 Hz, 1H). **^13^C NMR** (176 MHz, DMSO-*d*_6_) δ 173.2, 170.3, 167.5, 167.4, 167.2, 165.9, 155.5, 152.9, 147.8, 143.1, 142.1, 141.4, 141.1, 138.5, 137.4, 137.3, 133.5, 131.4, 130.0, 129.8, 127.2 (q, *J =* 31.5 Hz), 124.1 (q, *J =* 273.8 Hz), 122.4, 121.5, 120.8, 120.7, 117.2, 116.5, 115.8, 114.2, 70.2, 70.2, 70.1, 70.1, 69.3, 69.1, 68.0, 49.3, 40.9, 39.5, 39.4, 38.9, 31.4, 22.5. **^19^F NMR** (377 MHz, DMSO*d*_6_) δ -58.12, -74.33. **HRMS (ESI-MS)** calcd for C_44_H_44_F_3_N_10_O_11_^+^ m/z (M+H)^+^ 945.3138, found: 945.3148.

**HBC-13**

**(N-(4-(2-((2-(2,6-dioxopiperidin-3-yl)-1,3-dioxoisoindolin-4-yl)oxy)acetamido)butyl)-4-(3-(3-(1-methyl-1H-pyrazol-4-yl)quinoxalin-6-yl)ureido)-2-(trifluoromethyl)benzamide)**

Yield = 59%; **^1^H NMR** (499 MHz, MeOD) δ 9.03 (s, 1H), 8.46 (s, 1H), 8.28 (dd, *J =* 6.6, 1.5 Hz, 2H), 7.98 – 7.91 (m, 2H), 7.81 (dd, *J =* 8.5, 7.4 Hz, 1H), 7.75 (dt, *J =* 9.1, 2.6 Hz, 2H), 7.53 (d, *J =* 7.3 Hz, 1H), 7.45 (dd, *J =* 15.3, 8.4 Hz, 2H), 5.13 (dd, *J =* 12.6, 5.5 Hz, 1H), 4.01 (s, 3H), 3.38 (d, *J =* 6.2 Hz, 4H), 3.35 (s, 1H), 2.90 – 2.80 (m, 1H), 2.79 – 2.70 (m, 2H), 2.66 (s, 1H), 2.14 (ddd, *J =* 10.0, 5.6, 2.9 Hz, 1H), 1.75 – 1.62 (m, 4H). **^13^C NMR** (176 MHz, DMSO-*d*_6_) δ 173.2, 170.4, 167.3, 167.2, 167.2, 166.0, 155.6, 152.9, 147.8, 143.1, 142.1, 141.4, 141.0, 138.5, 137.4, 137.3, 133.5, 131.4, 130.0, 129.8, 127.1 (q, *J =* 32.5 Hz), 124.1 (q, *J =* 275.2 Hz), 122.4, 121.6, 120.9, 120.7, 117.3, 116.5, 115.8, 114.2, 68.1, 49.3, 40.9, 39.4, 39.2, 38.6, 31.4, 26.9, 26.7, 22.5. **^19^F NMR** (377 MHz, DMSO-*d*_6_) δ -58.12, -73.47. **HRMS (ESI-MS)** calcd for C_40_H_36_F_3_N_10_O_8_^+^ m/z (M+H)^+^ 841.2664, found: 841.2668.

**HBC-14**

**(N-(2-(2-((2-(2,6-dioxopiperidin-3-yl)-1,3-dioxoisoindolin-4-yl)oxy)acetamido)ethyl)-4-(3-(3-(1-methyl-1H-pyrazol-4-yl)quinoxalin-6-yl)ureido)-2-(trifluoromethyl)benzamide)**

Yield = 63%; **^1^H NMR** (499 MHz, MeOD) δ 9.05 (d, *J =* 2.9 Hz, 1H), 8.48 (s, 1H), 8.31 (d, *J =* 2.4 Hz, 1H), 8.29 (s, 1H), 7.96 (d, *J =* 9.0 Hz, 1H), 7.90 (d, *J =* 2.2 Hz, 1H), 7.82 – 7.71 (m, 3H), 7.55 – 7.49 (m, 2H), 7.45 (d, *J =* 8.5 Hz, 1H), 5.11 (dd, *J =* 12.7, 5.4 Hz, 1H), 4.80 (s, 3H), 4.02 (s, 3H), 3.56 (s, 3H), 3.35 (s, 2H), 2.87 (ddd, *J =* 16.7, 13.7, 5.7 Hz, 1H), 2.80 – 2.67 (m, 2H), 2.66 (s, 2H), 2.19 – 2.10 (m, 1H). **HRMS (ESI-MS)** calcd for C_38_H_32_F_3_N_10_O_8_+ m/z (M+H)^+^ 813.2351, found: 813.2352.

**HBC-15**

**(N-(2-((2-(2,6-dioxopiperidin-3-yl)-1,3-dioxoisoindolin-4-yl)oxy)ethyl)-4-(3-(3-(1-methyl-1H-pyrazol-4-yl)quinoxalin-6-yl)ureido)-2-(trifluoromethyl)benzamide)**

Yield = 85%; **^1^H NMR** (499 MHz, MeOD) δ 9.03 (s, 1H), 8.46 (s, 1H), 8.30 – 8.24 (m, 2H), 7.97 – 7.90 (m, 2H), 7.84 – 7.72 (m, 3H), 7.57 – 7.47 (m, 3H), 5.11 (dd, *J =* 12.7, 5.5 Hz, 1H), 4.41 (t, *J =* 5.2 Hz, 2H), 4.01 (s, 3H), 3.82 (t, *J =* 5.2 Hz, 2H), 2.87 (ddd, *J =* 17.1, 13.8, 5.3 Hz, 1H), 2.79 – 2.67 (m, 2H), 2.66 (s, 1H), 2.13 (dtd, *J =* 13.1, 5.1, 2.2 Hz, 1H). **^13^C NMR** (176 MHz, DMSO-*d*_6_) δ 173.3, 170.4, 167.8, 167.3, 165.7, 156.2, 152.9, 147.8, 143.1, 142.1, 141.4, 141.2, 138.5, 137.6, 137.3, 133.8, 131.4, 130.2, 130.0, 129.8, 127.3 (q, *J =* 32.6 Hz), 124.1 (q, *J =* 274.1 Hz), 122.4, 121.5, 120.7, 117.1, 116.1, 115.9, 115.6, 114.2, 67.5, 49.2, 40.9, 39.0, 31.4, 22.5. **^19^F NMR** (377 MHz, DMSO-*d*_6_) δ -58.11, -74.46. **HRMS (ESI-MS)** calcd for C_36_H_29_F_3_N_9_O_7_^+^ m/z (M+H)^+^ 756.2137, found: 756.2137.

**HBC-16**

**(2-bromo-N-(2-(2-(2-((2-(2,6-dioxopiperidin-3-yl)-1,3-dioxoisoindolin-4-yl)amino)ethoxy)ethoxy)ethyl)-4-(3-(3-(1-methyl-1H-pyrazol-4-yl)quinoxalin-6-yl)ureido)benzamide)**

**^1^H NMR** (500 MHz, MeOD) δ 9.07 (s, 1H), 8.49 (s, 1H), 8.36 – 8.26 (m, 2H), 7.98 (d, *J =* 9.0 Hz, 1H), 7.87 (t, *J =* 1.1 Hz, 1H), 7.76 (dd, *J =* 9.0, 2.4 Hz, 1H), 7.55 (dd, *J =* 8.6, 7.1 Hz, 1H), 7.39 (d, *J =* 1.2 Hz, 2H), 7.06 (dd, *J =* 7.9, 3.9 Hz, 2H), 5.05 (dd, *J =* 12.8, 5.5 Hz, 1H), 4.04 (s, 3H), 3.77 (t, *J =* 5.2 Hz, 2H), 3.72 (d, *J =* 4.4 Hz, 5H), 3.58 (t, *J =* 5.3 Hz, 2H), 3.48 (d, *J =* 5.3 Hz, 2H), 2.92 – 2.81 (m, 1H), 2.78 – 2.68 (m, 2H), 2.18 – 2.07 (m, 1H). **HRMS (ESI-MS)** calcd for C_39_H_38_BrN_10_O_8_^+^ m/z (M+H)^+^ 853.2052, found: 853.2054.

**HBC-17**

**(2-bromo-N-(14-((2-(2,6-dioxopiperidin-3-yl)-1,3-dioxoisoindolin-4-yl)amino)-3,6,9,12-tetraoxatetradecyl)-4-(3-(3-(1-methyl-1H-pyrazol-4-yl)quinoxalin-6-yl)ureido)benzamide)**

**^1^H NMR** (500 MHz, MeOD) δ 9.05 (s, 1H), 8.48 (s, 1H), 8.30 (d, *J =* 2.3 Hz, 2H), 8.00 – 7.91 (m, 2H), 7.74 (dd, *J =* 9.0, 2.4 Hz, 1H), 7.54 – 7.40 (m, 3H), 7.01 (dd, *J =* 18.1, 7.8 Hz, 2H), 5.04 (d, *J =* 5.5 Hz, 1H), 4.04 (s, 3H), 3.72 (t, *J =* 5.3 Hz, 2H), 3.69 – 3.62 (m, 14H), 3.56 (t, *J =* 5.4 Hz, 2H), 3.50 – 3.46 (m, 2H), 2.91 – 2.81 (m, 1H), 2.79 – 2.69 (m, 2H), 2.18 – 2.07 (m, 1H). **^13^C NMR** (176 MHz, DMSO) δ 172.8, 170.1, 168.9, 167.3, 167.0, 152.2, 147.4, 146.4, 142.7, 141.6, 141.3, 140.9, 138.0, 136.8, 136.2, 132.3, 132.1, 130.9, 129.4, 129.3, 121.9, 121.6, 120.2, 119.3, 117.4, 116.9, 113.6, 110.7, 109.2, 69.8, 69.8, 69.8, 69.6, 68.9, 68.7, 48.5, 41.7, 40.4, 40.0, 39.0, 38.9, 31.0, 22.1. **HRMS (ESI-MS)** calcd for C_43_H_46_BrN_10_O_10_^+^ m/z (M+H)^+^ 941.2576, found: 941.2576.

**HBC-18**

**(2-bromo-N-(1-((2-(2,6-dioxopiperidin-3-yl)-1,3-dioxoisoindolin-4-yl)oxy)-2-oxo-6,9,12-trioxa-3-azatetradecan-14-yl)-4-(3-(3-(1-methyl-1H-pyrazol-4-yl)quinoxalin-6-yl)ureido)benzamide)**

**^1^H NMR** (500 MHz, MeOD) δ 9.05 (s, 1H), 8.49 (s, 1H), 8.31 – 8.26 (m, 2H), 7.99 – 7.89 (m, 2H), 7.75 – 7.69 (m, 2H), 7.50 (dd, *J =* 8.3, 2.1 Hz, 1H), 7.47 – 7.40 (m, 2H), 7.34 (d, *J =* 8.4 Hz, 1H), 5.15 – 5.10 (m, 1H), 4.74 (s, 2H), 4.04 (s, 3H), 3.70 – 3.65 (m, 8H), 3.62 (dd, *J =* 12.2, 5.7 Hz, 4H), 3.56 (t, *J =* 5.3 Hz, 2H), 3.50 (t, *J =* 5.5 Hz, 2H), 2.93 – 2.82 (m, 1H), 2.81 – 2.70 (m, 2H), 2.17 (dd, *J =* 10.4, 2.3 Hz, 1H). **^13^C NMR** (176 MHz, DMSO) δ 172.8, 169.9, 167.1, 166.9, 166.7, 165.4, 155.0, 152.2, 147.4, 142.6, 141.6, 141.3, 140.9, 138.0, 136.9, 136.8, 133.0, 132.3, 131.0, 129.4, 129.3, 121.9, 121.6, 120.3, 120.2, 119.3, 116.9, 116.8, 116.0, 113.6, 69.8, 69.7, 69.6, 69.6, 68.8, 68.7, 67.5, 48.8, 40.4, 40.0, 38.4, 30.9, 22.0. **HRMS (ESI-MS)** calcd for C_43_H_44_BrN_10_O_11_^+^ m/z (M+H)^+^ 955.2369, found: 955.2366.

**HBC-19**

**(2-bromo-N-(4-(2-((2-(2,6-dioxopiperidin-3-yl)-1,3-dioxoisoindolin-4-yl)oxy)acetamido)butyl)-4-(3-(3-(1-methyl-1H-pyrazol-4-yl)quinoxalin-6-yl)ureido)benzamide)**

**^1^H NMR** (500 MHz, MeOD) δ 9.06 (s, 1H), 8.49 (s, 1H), 8.34 – 8.28 (m, 2H), 7.97 (d, *J =* 9.0 Hz, 1H), 7.93 (d, *J =* 2.1 Hz, 1H), 7.84 (dd, *J =* 8.4, 7.4 Hz, 1H), 7.77 (dd, *J =* 9.0, 2.4 Hz, 1H), 7.56 (d, *J =* 7.3 Hz, 1H), 7.51 – 7.44 (m, 2H), 7.38 (d, *J =* 8.3 Hz, 1H), 5.15 (dd, *J =* 12.7, 5.4 Hz, 1H), 4.80 (s, 2H), 4.04 (s, 3H), 3.42 (s, 4H), 2.93 – 2.82 (m, 1H), 2.81 – 2.71 (m, 2H), 2.17 (d, *J =* 4.9 Hz, 1H), 1.74 (s, 4H). **^13^C NMR** (176 MHz, DMSO) δ 172.8, 169.9, 166.9, 166.7, 166.7, 165.5, 155.1, 152.3, 147.4, 142.7, 141.6, 141.2, 141.0, 138.0, 137.0, 136.8, 133.0, 132.7, 131.0, 129.3, 121.9, 121.6, 120.4, 120.2, 119.3, 117.0, 116.8, 116.0, 113.6, 67.7, 48.8, 40.4, 38.6, 38.1, 30.9, 26.6, 26.3, 22.0. **HRMS (ESI-MS)** calcd for C_39_H_36_BrN_10_O_8_^+^ m/z (M+H)^+^ 851.1895, found: 851.1895.

**HBC-20**

**(2-bromo-N-(2-(2-((2-(2,6-dioxopiperidin-3-yl)-1,3-dioxoisoindolin-4-yl)amino)ethoxy)ethyl)-4-(3-(3-(1-methyl-1H-pyrazol-4-yl)quinoxalin-6-yl)ureido)benzamide)**

**^1^H NMR** (500 MHz, MeOD) δ 9.06 (s, 1H), 8.49 (s, 1H), 8.31 (dd, *J =* 8.0, 1.5 Hz, 2H), 7.97 (d, *J =* 9.0 Hz, 1H), 7.88 (d, *J =* 2.1 Hz, 1H), 7.76 (dd, *J =* 9.0, 2.4 Hz, 1H), 7.54 (dd, *J =* 8.6, 7.1 Hz, 1H), 7.47 (dd, *J =* 8.4, 2.1 Hz, 1H), 7.36 (d, *J =* 8.3 Hz, 1H), 7.13 (d, *J =* 8.5 Hz, 1H), 7.02 (d, *J =* 7.0 Hz, 1H), 5.03 (dd, *J =* 12.7, 5.5 Hz, 1H), 4.04 (s, 3H), 3.78 (t, *J =* 5.2 Hz, 2H), 3.73 (t, *J =* 5.3 Hz, 2H), 3.65 – 3.51 (m, 4H), 2.93 – 2.84 (m, 1H), 2.80 – 2.68 (m, 2H), 2.19 – 2.11 (m, 1H).

**HRMS (ESI-MS)** calcd for C_37_H_34_BrN_10_O_7_^+^ m/z (M+H)^+^ 809.1790, found: 809.1795.

**HBC-21**

**(2-bromo-N-(2-(2-(2-(2-((2-(2,6-dioxopiperidin-3-yl)-1,3-dioxoisoindolin-4-yl)amino)ethoxy)ethoxy)ethoxy)ethyl)-4-(3-(3-(1-methyl-1H-pyrazol-4-yl)quinoxalin-6-yl)ureido)benzamide)**

**^1^H NMR** (500 MHz, MeOD) δ 9.06 (s, 1H), 8.48 (s, 1H), 8.34 – 8.25 (m, 2H), 7.96 (d, *J =* 9.0 Hz, 1H), 7.92 (d, *J =* 2.0 Hz, 1H), 7.73 (dd, *J =* 9.1, 2.4 Hz, 1H), 7.55 – 7.45 (m, 2H), 7.41 (d, *J =* 8.4 Hz, 1H), 7.06 (d, *J =* 8.6 Hz, 1H), 7.01 (d, *J =* 7.1 Hz, 1H), 5.05 (dd, *J =* 12.7, 5.5 Hz, 1H), 4.04 (s, 3H), 3.74 – 3.63 (m, 12H), 3.56 (t, *J =* 5.3 Hz, 2H), 3.48 (t, *J =* 5.3 Hz, 2H), 2.91 – 2.80 (m, 1H), 2.80 – 2.69 (m, 2H), 2.16 – 2.08 (m, 1H). **^13^C NMR** (176 MHz, DMSO) δ 172.8, 170.1, 168.9, 167.3, 167.0, 152.2, 147.4, 146.4, 142.7, 141.6, 141.3, 140.9, 138.0, 136.8, 136.2, 132.3, 132.1, 131.0, 129.4, 129.3, 121.9, 121.6, 120.2, 119.3, 117.5, 116.9, 113.6, 110.7, 109.2, 69.8, 69.8, 69.8, 69.6, 68.9, 68.7, 48.6, 41.7, 38.9, 31.0, 22.1. **HRMS (ESI-MS)** calcd for C_41_H_42_BrN_10_O_9_^+^ m/z (M+H)^+^ 897.2314, found: 897.2313.

**HBC-22**

**(2-bromo-N-(2-((2-(2,6-dioxopiperidin-3-yl)-1,3-dioxoisoindolin-4-yl)amino)ethyl)-4-(3-(3-(1-methyl-1H-pyrazol-4-yl)quinoxalin-6-yl)ureido)benzamide)**

**^1^H NMR** (500 MHz, MeOD) δ 9.05 (s, 1H), 8.48 (s, 1H), 8.29 (d, *J =* 2.4 Hz, 2H), 8.00 – 7.91 (m, 2H), 7.76 (dd, *J =* 9.0, 2.4 Hz, 1H), 7.61 (dd, *J =* 8.6, 7.1 Hz, 1H), 7.48 (dd, *J =* 8.4, 2.1 Hz, 1H), 7.37 (d, *J =* 8.4 Hz, 1H), 7.23 (d, *J =* 8.5 Hz, 1H), 7.10 (d, *J =* 7.0 Hz, 1H), 5.09 (dd, *J =* 12.4, 5.5 Hz, 1H), 4.03 (s, 3H), 3.65 (d, *J =* 4.1 Hz, 4H), 2.94 – 2.83 (m, 1H), 2.81 – 2.70 (m, 2H), 2.18 – 2.09 (m, 1H). **HRMS (ESI-MS)** calcd for C_35_H_30_BrN_10_O_6_^+^ m/z (M+H)^+^ 765.1528, found: 765.1530.

**HBC-23**

**(2-bromo-N-(3-((2-(2,6-dioxopiperidin-3-yl)-1,3-dioxoisoindolin-4-yl)amino)propyl)-4-(3-(3-(1-methyl-1H-pyrazol-4-yl)quinoxalin-6-yl)ureido)benzamide)**

**^1^H NMR** (500 MHz, MeOD) δ 9.06 (s, 1H), 8.48 (s, 1H), 8.30 (d, *J =* 2.8 Hz, 2H), 7.99 – 7.93 (m, 2H), 7.77 (dd, *J =* 9.2, 2.4 Hz, 1H), 7.63 – 7.57 (m, 1H), 7.51 (dd, *J =* 8.4, 2.1 Hz, 1H), 7.40 (d, *J =* 8.4 Hz, 1H), 7.13 (d, *J =* 8.6 Hz, 1H), 7.08 (d, *J =* 7.0 Hz, 1H), 5.08 (dd, *J =* 12.4, 5.4 Hz, 1H), 4.04 (s, 3H), 3.52 (t, *J =* 6.6 Hz, 3H), 2.93 – 2.84 (m, 1H), 2.81 – 2.72 (m, 2H), 2.18 – 2.09 (m, 1H), 2.03 – 1.98 (m, 1H). **^13^C NMR** (101 MHz, DMSO) δ 172.8, 170.1, 168.8, 167.3, 167.1, 152.2, 147.4, 146.3, 142.7, 141.6, 141.3, 140.9, 138.0, 136.8, 136.3, 132.6, 132.3, 130.9, 129.4, 129.3, 121.9, 121.6, 120.2, 119.3, 117.2, 117.0, 113.6, 110.4, 109.2, 79.2, 48.5, 36.4, 31.0, 30.7, 28.6, 22.2. **HRMS (ESI-MS)** calcd for C_36_H_32_BrN_10_O_6_^+^ m/z (M+H)^+^ 779.1684, found: 779.1684.

**References**

1.	van de Wetering M, Francies HE, Francis JM, Bounova G, Iorio F, Pronk A, et al. Prospective derivation of a living organoid biobank of colorectal cancer patients. Cell. 2015;161(4):933–45. <https://doi.org/10.1016/j.cell.2015.03.053>

2.	Corsello SM, Nagari RT, Spangler RD, Rossen J, Kocak M, Bryan JG, et al. Discovering the anti-cancer potential of non-oncology drugs by systematic viability profiling. Nat Cancer. 2020;1(2):235–48. <https://doi.org/10.1038/s43018-019-0018-6>

3.	Perez-Riverol Y, Bandla C, Kundu DJ, Kamatchinathan S, Bai J, Hewapathirana S, et al. The PRIDE database at 20 years: 2025 update. Nucleic Acids Res. 2025;53(D1):D543–D53. <https://doi.org/10.1093/nar/gkae1011>

4.	Napoleon JV, Sagar S, Kubica SP, Boghean L, Kour S, King HM, et al. Small-molecule IKKbeta activation modulator (IKAM) targets MAP3K1 and inhibits pancreatic tumor growth. Proc Natl Acad Sci U S A. 2022;119(18):e2115071119. <https://doi.org/10.1073/pnas.2115071119>

5.	Boghean L, Singh S, Mangalaparthi KK, Kizhake S, Umeta L, Wishka D, et al. A Selective MAP3K1 Inhibitor Facilitates Discovery of NPM1 as a Member of the Network. Molecules. 2025;30(9). <https://doi.org/10.3390/molecules30092001>
